# Retinol-binding protein 4 (RBP4) circulating levels and gestational diabetes mellitus: a systematic review and meta-analysis

**DOI:** 10.3389/fpubh.2024.1348970

**Published:** 2024-03-12

**Authors:** Bianca M. Leca, Chris Kite, Lukasz Lagojda, Allan Davasgaium, Alex Dallaway, Kamaljit Kaur Chatha, Harpal S. Randeva, Ioannis Kyrou

**Affiliations:** ^1^Warwickshire Institute for the Study of Diabetes, Endocrinology and Metabolism (WISDEM), University Hospitals Coventry and Warwickshire NHS Trust, Coventry, United Kingdom; ^2^Warwick Medical School, University of Warwick, Coventry, United Kingdom; ^3^School of Health and Society, Faculty of Education, Health and Wellbeing, University of Wolverhampton, Wolverhampton, United Kingdom; ^4^Centre for Sport, Exercise and Life Sciences, Research Institute for Health and Wellbeing, Coventry University, Coventry, United Kingdom; ^5^Chester Medical School, University of Chester, Shrewsbury, United Kingdom; ^6^Clinical Evidence-Based Information Service (CEBIS), University Hospitals Coventry and Warwickshire NHS Trust, Coventry, United Kingdom; ^7^Department of Biochemistry and Immunology, University Hospitals Coventry and Warwickshire NHS Trust, Coventry, United Kingdom; ^8^Institute for Cardiometabolic Medicine, University Hospitals Coventry and Warwickshire NHS Trust, Coventry, United Kingdom; ^9^Aston Medical School, College of Health and Life Sciences, Aston University, Birmingham, United Kingdom; ^10^College of Health, Psychology and Social Care, University of Derby, Derby, United Kingdom; ^11^Laboratory of Dietetics and Quality of Life, Department of Food Science and Human Nutrition, School of Food and Nutritional Sciences, Agricultural University of Athens, Athens, Greece

**Keywords:** retinol-binding protein 4, RBP4, gestational diabetes mellitus, GDM, pregnancy, systematic review, meta-analysis

## Abstract

**Background:**

Gestational diabetes mellitus (GDM) is a prevalent condition where diabetes is diagnosed during pregnancy, affecting both maternal and fetal outcomes. Retinol-binding protein 4 (RBP4) is a circulating adipokine which belongs to the lipocalin family and acts as a specific carrier protein that delivers retinol (vitamin A) from the liver to the peripheral tissues. Growing data indicate that circulating RBP4 levels may positively correlate with GDM. Thus, this systematic review and meta-analysis aimed to investigate the potential relationship between circulating RBP4 levels and GDM when measured at various stages of pregnancy.

**Methods:**

MEDLINE, CINAHL, EMCARE, EMBASE, Scopus, and Web of Science databases were searched to identify studies comparing pregnant women with and without GDM, whose circulating RBP4 levels were measured in at least one pregnancy trimester. Findings were reported using standardized mean difference (SMD) and random-effects models were used to account for variability among studies. Furthermore, the risk of bias was assessed using the RoBANS tool.

**Results:**

Out of the 34 studies identified, 32 were included in the meta-analysis (seven with circulating RBP4 levels measured in the first trimester, 19 at 24–28 weeks, and 14 at >28 weeks of pregnancy). RBP4 levels were statistically higher in the GDM group than in controls when measured during all these pregnancy stages, with the noted RBP4 SMD being 0.322 in the first trimester (95% CI: 0.126–0.517; *p* < 0.001; 946 GDM cases vs. 1701 non-GDM controls); 0.628 at 24–28 weeks of gestation (95% CI: 0.290–0.966; *p* < 0.001; 1776 GDM cases vs. 1942 controls); and 0.875 at >28 weeks of gestation (95% CI: 0.252–1.498; *p* = 0.006; 870 GDM cases vs. 1942 non-GDM controls). Significant study heterogeneity was noted for all three pregnancy timepoints.

**Conclusion:**

The present findings indicate consistently higher circulating RBP4 levels in GDM cases compared to non-GDM controls, suggesting the potential relevance of RBP4 as a biomarker for GDM. However, the documented substantial study heterogeneity, alongside imprecision in effect estimates, underscores the need for further research and standardization of measurement methods to elucidate whether RBP4 can be utilized in clinical practice as a potential GDM biomarker.

**Systematic review registration:**

PROSPERO (CRD42022340097: https://www.crd.york.ac.uk/prospero/display_record.php?ID=CRD42022340097).

## 1 Introduction

Diabetes diagnosed during pregnancy, i.e., gestational diabetes mellitus (GDM), is a highly prevalent condition that is typically characterized by hyperglycemia, glucose intolerance, and insulin resistance, potentially resulting in adverse effects for both the mother and the fetus (1). The reported GDM prevalence rates range from 1 to 14% depending on the studied population, with Asia, Latin America, and the Middle East regions exhibiting higher prevalence rates, whilst inconsistencies in the testing protocols and diagnostic criteria further contribute to the varying GDM prevalence rates reported worldwide (2). In the United Kingdom, approximately 1 in 23 pregnancies is affected by GDM (3). GDM frequently resolves soon after delivery, but these women are more likely to experience GDM in subsequent pregnancies and have an increased risk of later developing type 2 diabetes (4, 5).

Several factors contribute to a higher risk of developing GDM, including an increased body mass index (BMI) at overweight or obesity levels, excessive weight gain during pregnancy, specific ethnic backgrounds (e.g., women from South Asia), genetic factors, a personal or family history of GDM, and polycystic ovary syndrome (PCOS) (6–8). Currently, to diagnose GDM, most pregnant women are offered an oral glucose tolerance test (OGTT) between 24 and 28 weeks of gestation or earlier for those considered at high risk (9). However, using pre-diagnostic risk factor screening alone is not always an effective method of identifying women at risk of GDM, as shown by meta-analysis data (9). This highlights that there is still a need for novel biomarkers to more accurately identify women at high GDM risk. As such, recent research in the field of GDM has focused on studying an array of biomarkers which can be measured in the circulation of pregnant women and are linked to the complex pathophysiology of the condition, such as biomarkers associated with obesity-related inflammation, insulin resistance, and those derived from the adipose tissue (i.e., adipokines) or the placenta.

Retinol-binding protein 4 (RBP4) is a 21-kDa protein (10), which is secreted mainly by the liver and adipose tissue, and was initially identified as a transport protein for retinol (vitamin A) and other retinoid derivatives in the bloodstream (11). A 2005 study showed for the first time the potential involvement of RBP4 in the pathogenesis of type 2 diabetes (11), with the expression of RBP4 playing a regulatory role in glucose metabolism in both the liver and skeletal muscle. Indeed, the decreased expression of glucose transporter-4 (GLUT4) is linked to increased RBP4 secretion from the adipose tissue, which leads to increased hepatic gluconeogenesis and reduced glucose uptake in the muscle, ultimately resulting in increased blood glucose levels, impaired glucose tolerance, and diabetes (12). Furthermore, recent studies have also revealed close associations between RBP4 and cardiovascular disease (CVD) and related risk factors, such as obesity, hypertension, dyslipidemia, heart failure, and coronary heart disease (10).

In this context, there has been increasing interest in investigating the potential role of RBP4 as a novel biomarker for GDM. However, the reported results have been inconsistent, with previous meta-analyses suggesting that serum RBP4 levels in early pregnancy show an independent positive association with GDM risk (13), and that Asian women with GDM had increased circulating RBP4 levels during the second/third pregnancy trimester (14). Although such data support the hypothesis that circulating RBP4 may be linked to GDM (15), there is still a need for a comprehensive systematic analysis and an updated meta-analysis of the relevant published studies examining the association between GDM and circulating RBP4 levels measured during all pregnancy stages/trimesters. Therefore, the present systematic review and meta-analysis aimed to explore this potential relationship across the pregnancy duration, providing an up-to-date critical synthesis of the relevant available data.

## 2 Materials and methods

The present systematic review adhered to the Preferred Reporting Items for Systematic Reviews and Meta-analyses (PRISMA) (16) guidelines (Supplementary Table S1.1), and was prospectively registered on PROSPERO (International Prospective Register of Systematic Reviews – University of York), with the registration number CRD42022340097.

### 2.1 Search strategy and data sources

A search was conducted based on a predefined search strategy and was adapted to the syntax and appropriate subject headings of the following databases: MEDLINE, CINAHL, EMCARE, EMBASE via Ovid, Scopus, and Web of Science. Reference lists were also browsed to ensure literature saturation. Final searches were completed in June 2023, and the main search strategy for MEDLINE is presented in Table 1, whilst all other search strategies are detailed in Supplementary data and Supplementary material 1.2.

**Table 1:** MEDLINE search string.

(Retinol binding proteins[MeSH Terms]) OR (retinol binding protein 4) OR (retinol-binding protein-4) OR (retinol binding protein-4) OR RBP4 OR (RBP 4) OR (RBP-4)) AND (Pregnancy[MeSH Terms]) OR pregnan*))

### 2.2 Eligibility criteria

Eligible articles included those conducted in adult (age > 18 years old) pregnant women with and without GDM, whose circulating levels of RBP4 were measured during at least one pregnancy trimester. No restrictions were imposed regarding the year of publication, type of setting, language, or timing of RBP4 measurement during the pregnancy. All observational study designs were included, while single case reports, expert opinion manuscripts, commentaries, animal studies, and review articles were excluded.

### 2.3 Study selection and data extraction

The study selection and data extraction processes were conducted independently by two reviewers (BML and LL), and any discrepancies or disagreements were resolved through consultation with a third reviewer (CK).

The initial selection of potentially eligible studies was based on title and abstract screening and was performed using the Rayyan software (17), following a predefined protocol. Papers considered eligible progressed to a full-text review.

A standardized data extraction form was developed to extract relevant information from the included eligible studies. The extracted data included country of origin, study design, patient demographics, number of participants, and relevant study outcomes/findings (e.g., circulating RBP4 levels). In addition, attempts were made to contact the corresponding study investigators in cases where relevant data on circulating RBP4 levels were missing or reported as median and interquartile range (IQR). Where relevant responses were not received (18–25), median and IQR data were transformed using the formulas provided by Luo et al. (26) and Wan et al. (27). Furthermore, for one study (28) these values were extracted from figures using a plot digitizer,^1^ as previously reported (29).

Herein, data on circulating RBP4 levels are reported as mean and standard deviations (SDs) (30). For certain included studies (25, 31–33), it was necessary to combine study groups; this was done using recommended formulae (34).

When multiple methods were used to measure circulating RBP4 levels (23, 24), the enzyme linked immunosorbent assay (ELISA) result was chosen as the most commonly utilized method. Additionally, for Tepper et al. (35), a sensitivity analysis was conducted by switching the data to Western Blot due to the differences observed between measurements.

### 2.4 Quality assessment

The risk of bias for each included study was assessed independently by two reviewers (BML and LL) using the Risk of Bias Assessment Tool for Nonrandomized Studies (RoBANS) (36), which covers six domains, namely: selection of participants, confounding variables, exposure measurement, blinding of outcome assessment, incomplete outcome data, and selective outcome reporting. For each domain, the risk of bias was assessed as low, high, or unclear. Any disagreements were resolved through discussion between reviewers and if needed, consultation with a third reviewer (CK).

### 2.5 Statistical analysis

The statistical analysis was performed using Comprehensive Meta-Analysis Version 4.0 (37). The results were reported using the standardized mean difference (SMD) to quantify the magnitude of the effect and 95% confidence intervals (CI) as a measure of precision around effect estimates. The effect size represents the SMD between circulating RBP4 levels in the GDM group and the pregnant control group at different timepoints (i.e., at the first trimester, 24–28 weeks of gestation, and > 28 weeks of gestation).

A random-effects model was used for the performed meta-analysis, and the effect size for each timepoint was calculated. Heterogeneity among studies was assessed using Cochran’s *Q* and *I*^2^ statistics, and was considered significant if *p* < 0.1 in the *Q*-test whilst for the *I*^2^: 0–40% heterogeneity might not be important; 30–60% may represent moderate heterogeneity; 50–90% may represent substantial heterogeneity; and 75–100% represents considerable heterogeneity (30).

To investigate heterogeneity, we sub-grouped studies based upon the country in which they were conducted, the diagnostic criteria used to identify GDM cases, and the RBP4 measurement method/assay. It was not possible to sub-group based upon any other variable due to the incompleteness of reporting. Supplementary Table S2.1 presents the summary of effect estimates and heterogeneity for the sub-groups at each pregnancy stage.

For the studies where mean and SDs were calculated (18–25), sensitivity analysis was performed, removing studies that contained data significantly skewed away from the normal distribution (19, 21, 22, 24).

Where analyses included ten or more studies (30), publication bias was assessed using the Egger’s test and regression intercept. Additionally, a Duval and Tweedie’s trim-and-fill analysis was conducted to obtain an adjusted summary effect that accounts for publication bias.

## 3 Results

### 3.1 Study selection

**Figure 1:**
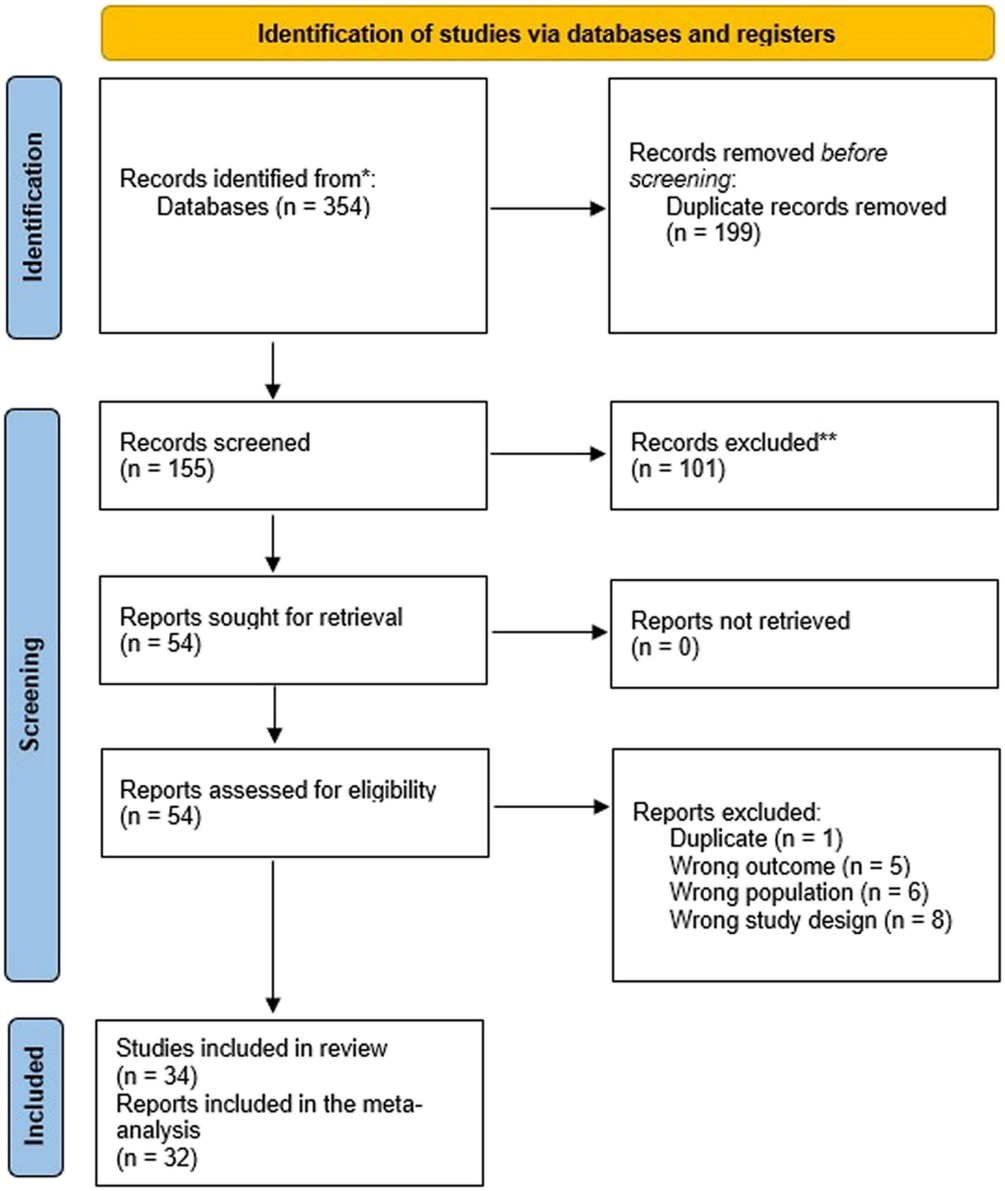
PRISMA flow diagram of the present systematic review.

A total of 354 articles were initially identified from the searched databases. Following deduplication in RefWorks, this number was refined to 155 unique records that required screening. Out of these, 101 records were excluded during the title and abstract screening process. The remaining 54 were successfully retrieved and the full texts were assessed for eligibility, resulting in the exclusion of 20 reports for various reasons, i.e., one was a duplicate, five had the wrong outcome, six involved the wrong population, and eight had the wrong study design (Figure 1). Furthermore, two studies (38, 39) were included in the review, but excluded from the meta-analysis because the reported data on RBP4 levels could not be extracted/converted for meta-analysis and repeated attempts to contact the authors were unsuccessful.

### 3.2 Risk of bias assessment

**Figure 2:**
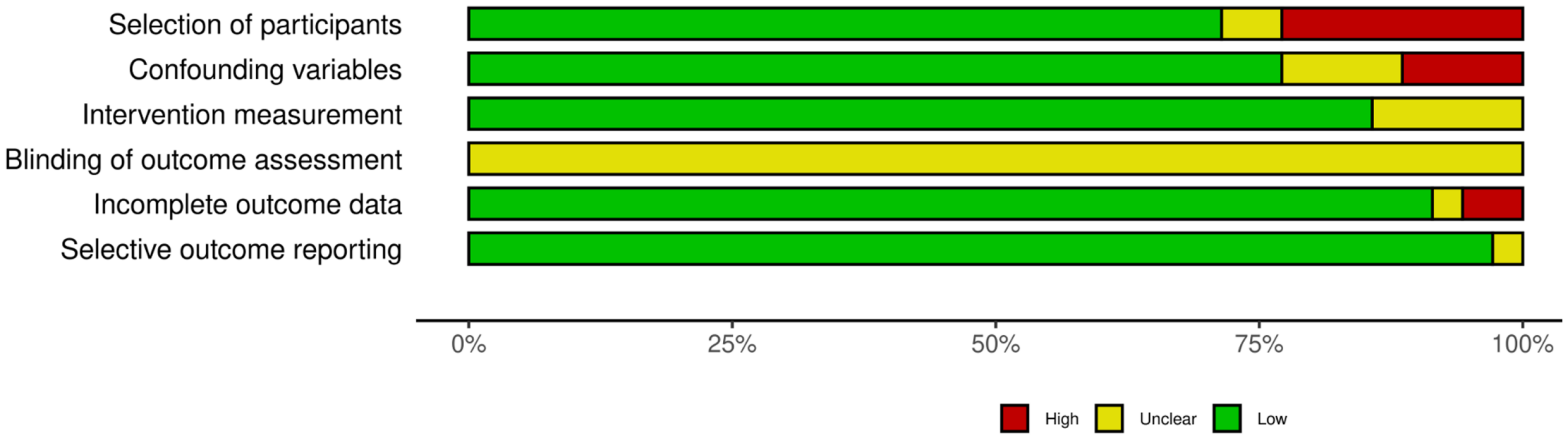
Risk of bias assessment - summary plot.

The risk of bias assessment of the included studies is presented in Figure 2 and in Supplementary Figure S2.2. Most studies (*n* = 24; 70.5%) had a low risk of bias in participant selection, although some lacked clarity in their selection methods (eight studies with high risk of bias, and two with unclear; Supplementary Figure S2.2). When it came to controlling for confounding variables, 27 studies (77%) were rated as having a low risk of bias, with four having an unclear risk, and three having a high risk in this regard. When assessing the exposure measurement, in five studies the exact criteria used to diagnose GDM were unclear, while the rest of the studies were classified as having a low risk of bias (87.1%). In terms of utilizing a valid measurement method for RBP4, 32 studies (94.1%) had a low risk of bias, but two had unclear measurement methods. Given that none of the studies were interventional, and therefore did not report on assessor blinding, all had an unclear risk of bias in blinding the outcome assessment. Concerning handling incomplete outcome data, one study was at a high risk of bias, while one other had an unclear risk in this category; the remaining studies (*n* = 32; 91%) were judged to have a low risk of bias. In the selective outcome reporting domain, all studies apart from one had a low risk of bias (13, 18–25, 28, 31–33, 35, 39–57); Zhu et al. (58) was judged to have an unclear risk.

### 3.3 Main characteristics of the included studies

The main characteristics of the included studies are presented in Table 2, and reported circulating RBP4 levels are presented in Supplementary Table S1.3. Of the 34 eligible studies, nine measured circulating RBP4 levels in the first trimester, 21 at 24–28 weeks, and 14 at >28 weeks of gestation. However, two studies did not report the measured RBP4 levels in a way that could be extracted (38, 39), so were not included in the meta-analysis. When sensitivity analyses were conducted by removing the studies with skewed data, the effect on estimates was negligible, therefore they were included in the analysis. The final selected studies included a total of 3,595 GDM cases and 4,544 non-GDM controls.

**Table 2:** General characteristics of the eligible studies included in the present systematic review and meta-analysis.

Authors (Country)	Group Characteristics	GDM diagnosis made by	Assay for RBP4	Unit	Data measured at (weeks of gestation)	Key outcome(s)
Abetew DF et al., 2013 (41), (United States)	GDM (*N* = 173, age = 34.15 ± 4.56); Controls (*N* = 187, age = 32.95 ± 4.32)	ADA	ELISA (Catalog number DRB400, Quantikine TM, R&D Systems, Minneapolis, MN, United States)	μg/mL	16	The mean serum RBP4 level was significantly higher among GDM cases than controls. There was modest evidence of a positive association of early pregnancy elevated RBP4 concentration with increased GDM risk, particularly among women of advanced age.
Chan TF et al., 2007 (42), (Taiwan)	GDM (*N* = 20, age = 32.7 ± 5, BMI = 26.1 ± 4.7); Controls (*N* = 20, age = 32.7 ± 5, BMI = 25.9 ± 2.9)	NDDG	ELISA (Immundiagnostik AG, Bensheim, Germany)	ng/mL	24–28, upon delivery	Serum RBP4 concentrations at glucose challenge test were significantly higher in the GDM group than in the healthy control group. BMI was significantly correlated to serum RBP4 concentrations by multiple linear regression analysis.
Chen and Du, 2011 (31), (China)	GDM (Obesity: age = 32 ± 4.8, normal weight: age = 31.7 ± 3.5, *N* = 52); Controls (Obesity: age = 28.4 ± 3.1, normal weight: age = 28.3 ± 3, *N* = 46)	N/A	ELISA	μg/L	37–39	Serum RBP4 levels were higher in obese pregnant women than in non-obese women. RBP4 levels in GDM with obesity were higher than in other groups.
Du M et al., 2016 (43), (China)	GDM (*N* = 38, age = 28.79 ± 4.04); Controls (*N* = 38, age = 28.92 ± 3.02)	NDDG	ELISA (R&D Company, United States)	μg/mL	37–42	RBP4 levels were higher in women with GDM. In healthy controls, RBP4 concentrations were positively correlated with HOMA-IR and TG.
Du X et al., 2019 (44), (China)	GDM (*N* = 194, age = 31.71 ± 3.63); Controls (*N* = 67, age = 31 ± 3.43)	FIGO	ELISA (R&D Systems in the United States of America)	μg/mL	24–28, 37–40	RBP4 levels were significantly higher in the GDM group compared to control group. RBP4 is related to GDM, and its levels increase with the increase of gestational weeks.
Ping F et al., 2012 (45), (China)	GDM (*N* = 488); GIGT (*N* = 235); NGT (*N* = 582); Normal (GCT−) (*N* = 290)	ADA	ELISA (Phoenix, Belmont, CA, United States EK-028-28)	μg/mL	13–15, 24–28	The estimated indices of IR gradually increased from NGT to GDM. RBP4 mRNA expression in adipose tissue of GDM patients was significantly increased.
Francis E et al., 2020 (39), (United States)	GDM (*N* = 107, age = 30.5 ± 5.7); Controls (*N* = 214, age = 30.4 ± 5.4)	Carpenter-Coustan	Quantikine Human RBP4 Immunoassay (R&D Systems)	N/A	10–14, 15–26, 23–31, 33–39	Adipokines, including FABP4, chemerin, and sOB-R may be implicated in the pathogenesis of GDM, with significant associations detected approximately 10–18 weeks before typical GDM screening. Chemerin and RBP4 were associated with a worse lipid profile.
Fruscalzo A et al., 2015 (32), (Italy)	GDM (iGDM: age = 33.55 ± 4.06, dGDM: age = 33.43 ± 4.03) (*N* = 32); Controls (AGA: age = 37.18 ± 4.44, LGA: age = 32.85 ± 3.47) (*N* = 64)	IADPSG	Non-commercial ELISA using polyclonal rabbit anti-human antibodies (Biozol, Eching, Germany)		11–13	GDM patients were characterised by reduced RBP4 compared to controls.
Gashlan H et al., 2017 (46), (Saudi Arabia)	GDM (*N* = 51, age = 32.4 ± 0.98, BMI = 33.8 ± 1.01); Controls (*N* = 37, age = 34 ± 1.52, BMI = 33.4 ± 0.81)	WHO	Assay from Elabscience Company (Wuhan, China)	ng/mL	2nd trimester, 3rd trimester	RBP4 was significantly decreased in GDM compared to control and was significantly correlated with IR in the GDM group only.
Gorkem U et al., 2016 (21), (Turkey)	GDM (*N* = 76, age = 29 (24–28), BMI = 33.25 (22.8–52.2)); Controls (*N* = 82, age = 26 (18–35), BMI = 26.43 (19.1–47))	Carpenter- Coustan	ELISA (Immundiagnostik, Immundiagnostik AG; Bensheim, Germany)	mg/mL	24–28	Serum RBP4 did not demonstrate significant differences between GDM and controls.
Hou W et al., 2018 (18), (China)	GDM (*N* = 131, age = 31.4 ± 3.8); Controls (*N* = 138, age = 30.4 ± 3.8)	IADPSG	N/A	mg/L	12	Multivariate models combining clinical markers and metabolites can potentially differentiate GDM subjects from healthy controls. Pre-pregnancy BMI was higher in GDM participants, as were ChE, RBP4, CysC and TG.
Jia X et al., 2022 (47), (China)	GDM (*N* = 62, age = 29.38 ± 4.65, BMI = 22.79 ± 2.93); Controls (*N* = 58, age = 28.93 ± 3.31, BMI = 25.8 ± 3.04)	People’s Republic of China Health Industry Standards	ELISA (American RD Company, San Francisco, CA, United States)	μg/mL	24–28	There were no statistically significant differences in RBP4 levels in GDM compared to healthy pregnancies. There were higher serum FGF-21 levels in GDM, which might be related to pre-pregnancy BMI, weight gain during pregnancy, leptin, RBP4, and adiponectin.
Jin C et al., 2020 (19), (China)	GDM (*N* = 135, age = 29 (28–33)); Controls (*N* = 135, age = 29 (28–33))	IADPSG	ELISA (R&D Systems China, Shanghai)	μg/L	< 14, 24–28	The GDM cases had significantly higher levels of RBP4 in the first trimester than controls. With adjustment for diet, physical activity, and other risk factors for GDM, the risk of GDM increased with every 1-log μg/L increment of RBP4 level.
Khovidhunkit W et al., 2012 (33), (Thailand)	GDM (*N* = 171, age = 33 (29–37)); Non-GDM (GIGT, NGT) (*N* = 361, age = 33 (28–36)); GCT− (*N* = 22, age = age = 32 (26–39))	Carpenter-Coustan	ELISA (R&D Systems Minneapolis, MN)	μg/mL	24–28	The degree of IR was higher in the GDM group than the non-GDM group, but serum RBP4 levels between the 2 groups were not different. Serum RBP4 levels in pregnancy are not associated with IR.
Kim SH et al., 2007 (48), (South Korea)	GDM (*N* = 10, age = 32.6 ± 3); Controls (*N* = 9, age = 32.6 ± 3.3)	ADA	ELISA (Immundiagnostik, Bensheim, Germany)	μg/mL	24–28	Women with GDM had higher RBP4 concentrations than those seen in healthy women during pregnancy, but short-term rise in serum insulin did not modulate circulating RBP4 concentrations.
Klein K et al., 2010 (49), (Austria)	GDM (*N* = 63, age = 33.3 ± 4.8, BMI = 28.1 ± 6.2); Controls (*N* = 38, age = 32.7 ± 5.2, BMI = 27.7 ± 5.6)	German & Austrian Society for Diabetes (modified Carpenter Coustan)	ELISA (DRG Instruments, Marburg, Germany)	mg/L	24–28, 33	Serum RBP4 levels increased significantly between the two measurements in patients with GDM. In patients with GDM, RBP4 concentrations at 33 weeks of gestation correlated positively with mean blood glucose and HbA1c values.
Krzyzanowska K et al., 2008 (24), (Austria)	GDM (*N* = 41, age = 33 (29–35), BMI = 34 (29–38); Controls (*N* = 45, age = 28 (24–34), BMI = 29 (25–31))	4th Workshop Conference of GDM	ELISA (RBP4 EIA kit; Phoenix Pharmaceuticals, Belmont, CA, United States)	μg/mL	29, 30, 8 weeks after delivery	Women with GDM had lower RBP4 levels than controls. The RBP4: retinol ratio and the RBP4:TTR ratio are more informative than RBP4 levels alone when assessing insulin–glucose homeostasis during pregnancy.
Kuzmicki M et al., 2011 (22), (Poland)	GDM (*N* = 88, age = 29.5 (27–33), BMI = 27.2 (25.2–30.1)); Controls (*N* = 86, age = 29.5 (27–31.5), BMI = 27.3 (23.1–29.4))	WHO	ELISA (Phoenix Pharmaceuticals, Inc., United States)	mg/L	24–30, 36–40	Serum RBP4 concentration and its expression in SAT were higher in the women with GDM than in the controls. No association between serum or tissue RBP4 and the indices of IR was noted.
Lewandowski KC et al., 2008 (50), (Poland)	GDM (GCT+, OGTT+) (*N* = 15, age = 34 (29–36), BMI = 26.3 (29.4–30.1)); IGT (GCT+, OGTT−) (*N* = 15, age = 32 (32–36), BMI = 25.1 (23.7–28.4)); Controls (GCT−, OGTT−) (*N* = 20, age = 32 (29–35), BMI = 25.1 (23.5–28.2))	WHO	Commercial RBP4 assay kit (Phoenix Pharmaceuticals Inc.: Burlingame, California, United States)	μg/mL	28	RBP4 levels were higher in women with GDM than in controls but did not correlate with IR.
Liu M et al., 2020 (51), (China)	GDM (*N* = 50, age = 33.88 ± 4.22, BMI = 27.69 ± 4.47); Controls (*N* = 47, age = 33.66 ± 3.97, BMI = 27.39 ± 2.32)	IADPSG	ELISA (Cusabio Biotech, Wuhan, Hubei, China)	μg/mL	37–42	GDM subjects had a lower RBP4/TTR ratio than the control subjects.
Maghbooli Z et al., 2010 (52), (Iran)	GDM (*N* = 92, age = 32.48 ± 5.23); Controls (*N* = 100, age = 27.88 ± 7.07)	O’Sullivan and Mahan criteria	ELISA (AdipoGen Kit, AdipoGen, Seoul, Korea)	μg/mL	24–28	RBP4 concentrations in GDM patients were significantly higher than in controls.
Mazaki-Tovi S et al., 2010 (25), (United States)	GDM (AGA: age = 34 (28–39), LGA: age = 32 (30–38)) (*N* = 97); Controls (AGA: age = 26 (22–29), LGA: age = 28 (22–32)) (*N* = 108)	WHO	Sensitive ELISA (Millipore Corporation, St. Charles, MO, United States)	ng/mL	>37	Patients with GDM had a higher median plasma concentration of RBP4 than normal pregnant women. GDM is characterized by alterations in maternal circulating RBP4 concentrations.
Nanda S et al., 2013 (20), (United Kingdom)	GDM (*N* = 60, age = 32 (28.5–35.6), BMI = 28.6 (24.6–4.2)); Controls (*N* = 240, age = 33 (27.3–35.9), BMI = 23.8 (21.7–26.2)); Pre-eclampsia (*N* = 60); LGA (*N* = 60); SGA (*N* = 60)	WHO	ELISA (Immundiagnostik, Stubenwaldallee, Bensheim, Germany	ng/mL	11–13	The serum concentration of RBP4 in the first trimester was not significantly different between the groups.
Ortega-Senovilla H et al., 2011 (28), (Spain)	GDM (*N* = 98, age = 30.9 ± 0.5); Controls (*N* = 86, age = 28.7 ± 0.5)	Carpenter- Coustan	Sandwich ELISA (AdipoGen, Seoul, Korea)	μg/mL	1 week before delivery	Maternal serum insulin, insulin-to-glucose ratio, HOMA-IR and RBP4 were higher, and adiponectin was lower in GDM than in control subjects.
Saucedo R et al., 2011 (40), (Mexico)	GDM (*N* = 60, age = 31.9 ± 5.6, BMI = 30.2 ± 4.9); Controls (*N* = 60, age = 24.8 ± 6.4, BMI = 28.4 ± 7.3)	ADA	RIA, using reagents from Phoenix Pharmaceuticals (Belmont, CA)	μg/mL	30, 6 weeks postpartum, 6 months postpartum	Women with GDM showed higher IR than controls. There was no difference in adipokines between the two groups, but in women with a healthy pregnancy, RBP4 was associated with IR.
Skvarca A et al., 2012 (23), (Slovenia)	GDM (*N* = 30, age = 30.33 ± 4.86, BMI = 27.57 (24.88–29.76)); IGT (*N* = 19, age = 30.84 ± 4.51, BMI = 27.61 (23.78–31.18)); Controls (*N* = 25, age = 31.2 ± 3.34, BMI = 25.39 (23.18–27.43))	4th Workshop Conference of GDM	Commercially available ELISA	mg/L	24–28	Significant differences in HOMA–IR were found, but no significant differences in serum adipokine levels. Adiponectin, leptin, resistin, visfatin and RBP4 were not associated with the degree of glucose intolerance in pregnancy.
Su YX et al., 2010 (53), (China)	NP-NGT (*N* = 65, age = 28.1 ± 3.4); GDM (*N* = 63, age = 28.8 ± 1.8, BMI = 25.5 ± 2.6); Controls (*N* = 58, age = 28.4 ± 2.4, BMI = 24.9 ± 2.1)	ADA	Sandwich ELISA (a protocol developed in-house) using affinity chromatography-purified polyclonal and monoclonal antibodies generated against recombinant human RBP4 protein	mg/L	24–28	Serum RBP4 levels in the pregnant NGT and GDM groups were significantly higher than in the non-pregnant. RBP4 levels were much higher in the GDM vs. pregnant NGT group. Serum RBP4 levels significantly increase in pregnancy, independent of age and BMI. RBP4 levels appear to be a valuable marker of IR and dysfunctional lipid metabolism in pregnancy.
Tepper BJ et al., 2010 (35), (United States)	GDM (*N* = 12, age = 28.6 ± 4.9, BMI = 31.1 ± 0.6); Controls (*N* = 10, age = 28.8 ± 6.2, BMI = 31.1 ± 0.9)	Carpenter-Coustan	ELISA	μmol/L	24–28	RBP4, retinol and RBP4/retinol molar ratio were not different between the groups; GDM is not associated with RBP4 or retinol among borderline-obese pregnant women.
Wu P et al., 2021 (13), (China)	GDM (*N* = 332, age = 28 (25–30)); Controls (*N* = 664, age = 28 (25–30))	IADPSG	ELISA (R&D Quantikine)	μg/mL	9–12	RBP4 was associated with a 1.39-fold higher risk of GDM. Serum RBP4 levels in early pregnancy, independent of metabolic risk factors, are positively associated with the risk of GDM.
Yuan X et al., 2017 (54), (China)	GDM (*N* = 86, age = 29 (27–33), BMI = 24.58 (21.72–26.98)); Controls (*N* = 273, age = 26 (24–28.25), BMI = 22.32 (20.66–28.25))	IADPSG	Automatic biochemical analyzer (Hitachi 7,180; Hitachi, Ibaraki-ken, Japan) using commercial kits (Wako Pure Chemical Industries, Osaka, Japan)	μg/mL	16–18	The group that developed GDM had statistically significantly higher concentrations of ficolin-3, CRP, RBP4 and FFAs than the control group. The elevated ratios of RBP4/adiponectin were also observed in participants who developed GDM.
Zhang H et al., 2022 (55), (China)	GDM (*N* = 70, age = 25.68 ± 4.27); Controls (*N* = 70, age = 27.02 ± 3.54)	Obstetrics and Gynecology Section of the Chinese Medical Association	ELISA (R&D System, United States)	mg/L	35–40	Glucose metabolism and islet function in women with GDM are significantly correlated with serum RBP4.
Zhang Y et al., 2016 (56), (China)	GDM (*N* = 40, age = 32.24 ± 3.81, BMI = 27.55 ± 3.4); Controls (*N* = 240, age = 28.21 ± 4.12, BMI = 24.31 ± 2.92)	IADPSG	ELISA (R&D Systems, China, Shanghai)	mg/L	24–28, >37	The GDM group showed greater levels of AFABP, leptin and RBP4 and a decreased adiponectin level.
Zhaoxia L et al., 2014 (57), (China)	GDM (*N* = 35, age = 29 ± 2.53); Controls (*N* = 35, age = 29.3 ± 3.06)	NDDG	Double antibody sandwich ELISA (Phoenix Pharmaceutical Company, Saint Joseph, MO)	μg/mL	24–28	Serum RBP4 levels in the GDM group were significantly higher than in the control group. Serum RBP4 levels in the GDM group were correlated with HOMA-IR, TG and blood glucose levels.
Zhu JP et al., 2014 (58), (China)	GDM (*N* = 177); Controls (*N* = 354)	N/A	N/A	mg/L	24–28	The plasma glucose, serum insulin, HOMA-IR, HbA1C and TG levels were significantly higher in the GDM group than in the controls. RBP4 levels of GDM women were significantly and positively correlated with the BMI.

Units: age: years; BMI: kg/m^2^. ADA, American Diabetes Association; AFABP, adipocyte fatty acid-binding protein; AGA, appropriate for gestational age; BMI, body mass index; ChE, chemerin E; CRP, C-reactive protein; CysC, cystatin C; dGDM, diet treated gestational diabetes mellitus; ELISA, enzyme-linked immunosorbent assay; FABP4, fatty acid binding protein 4; FGF-21, fibroblast growth factor 21; FIGO, International Federation of Gynecology and Obstetrics; GCT−, normal glucose challenge test; GCT+: abnormal glucose challenge test; GDM, gestational diabetes mellitus; GIGT, gestational impaired glucose tolerance; HbA1c, glycated hemoglobin; HOMA-IR, Homeostatic Model Assessment for Insulin Resistance; IADPSG, International Association of Diabetes and Pregnancy Study Groups; iGDM, insulin treated gestational diabetes mellitus; IGT, impaired glucose tolerance; IR, insulin resistance; LGA, large for gestational age; mRNA, messenger ribonucleic acid; NDDG, National Diabetes Data Group; NGT, normal glucose tolerance; OGTT−, normal oral glucose tolerance test; OGTT+, abnormal oral glucose tolerance test; PAI-1, plasminogen activator inhibitor-1; RBP4, retinol-binding protein 4; RIA, radioimmunoassay; SAT, subcutaneous adipose tissue; sOB-R, soluble leptin receptor; TG, triglycerides; TTR, transthyretin; WHO, World Health Organization.

### 3.4 Circulating RBP4 levels in the first trimester of pregnancy

**Figure 3:**
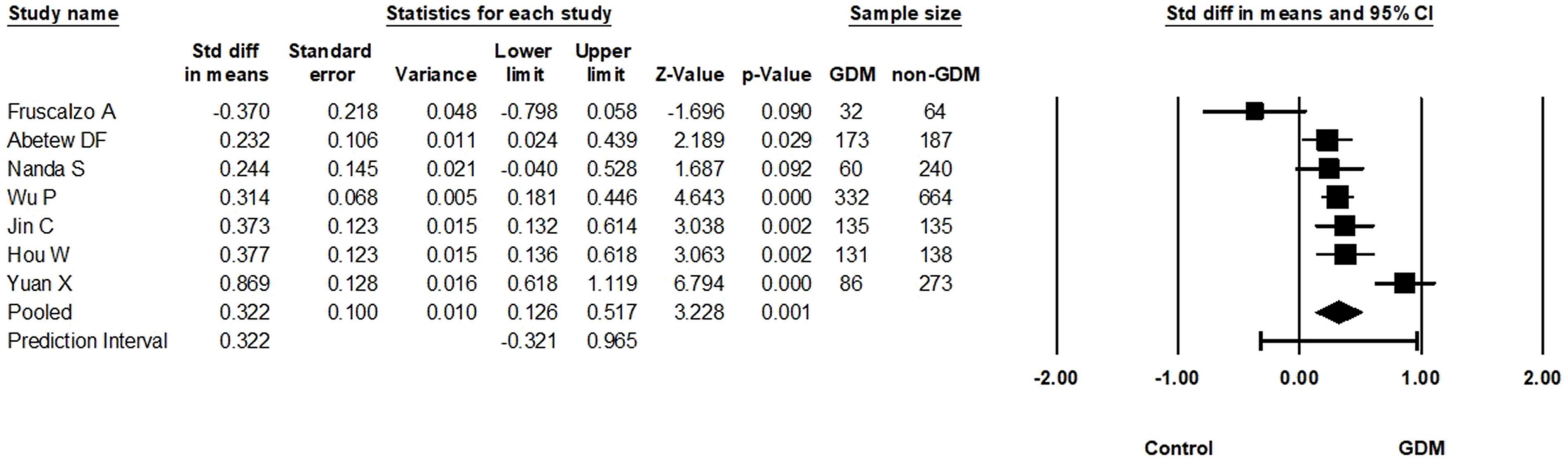
Forest plot of circulating RBP4 levels: gestational diabetes mellitus (GDM) compared to control in the first trimester of pregnancy. Std diff in means: standardized mean difference; CI: Confidence intervals.

From the nine studies that examined the relationship between circulating RBP4 levels during the first pregnancy trimester and GDM, seven were meta-analyzed (13, 18–20, 32, 41, 54) (946 GDM vs. 1701 non-GDM controls). Based on these, circulating RBP4 levels were statistically higher in pregnant women with GDM compared to pregnant controls (SMD: 0.322; 95% CI: 0.126 to 0.517; *p* < 0.001) (Figure 3). Moreover, there was substantial heterogeneity among these studies (*I*^2^ = 80%), although it is essential to acknowledge that the low number of eligible studies may limit the reliability of heterogeneity estimates (30). Additionally, removal of the study with skewed data in a sensitivity analysis (19) slightly reduced the SMD (0.309, 95% CI: 0.078–0.539; *p* = 0.009) (Supplementary Table S2.3).

### 3.5 Circulating RBP4 levels at 24–28 weeks of gestation

**Figure 4:**
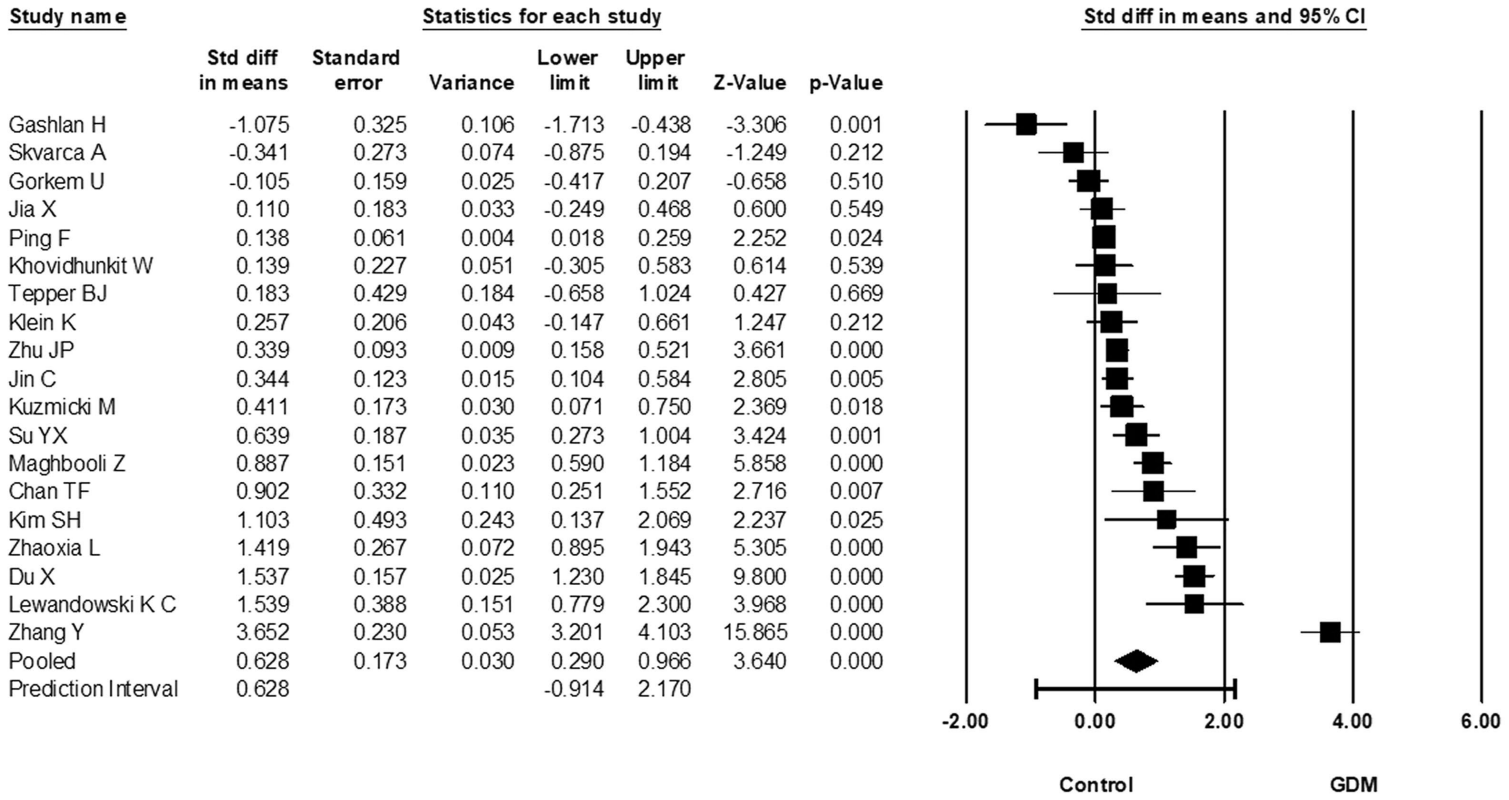
Forest plot of circulating RBP4 levels: gestational diabetes mellitus (GDM) compared to control at 24-28 weeks of gestation. Std diff in means: standardized mean difference; CI: Confidence intervals.

A total of 19 studies investigated the relationship between circulating RBP4 and GDM at 24–28 weeks of gestation and reported the corresponding RBP4 levels, with 1776 GDM cases and 1942 non-GDM controls in the performed meta-analysis. When compared to controls, circulating RBP4 levels at 24–28 weeks of gestation were significantly higher in women with GDM (SMD: 0.628; 95% CI: 0.290–0.966; *p* < 0.001) (Figure 4). Heterogeneity among these studies was considerable (*I*^2^ = 95%). Furthermore, when switching the reported RBP4 data from the Tepper et al. study (35) to their Western Blot reported data, the effect estimate remained similar at 0.620 (95% CI: 0.282–0.959; *p* < 0.001). Additionally, removal of the skewed studies (19, 21, 22) increased the effect size (SMD: 0.702; 95% CI: 0.289–1.115; *p* = 0.001). A one-study-removed analysis was also performed, as presented in Supplementary Figure S2.4.

### 3.6 Circulating RBP4 levels at more than 28 weeks of gestation

**Figure 5:**
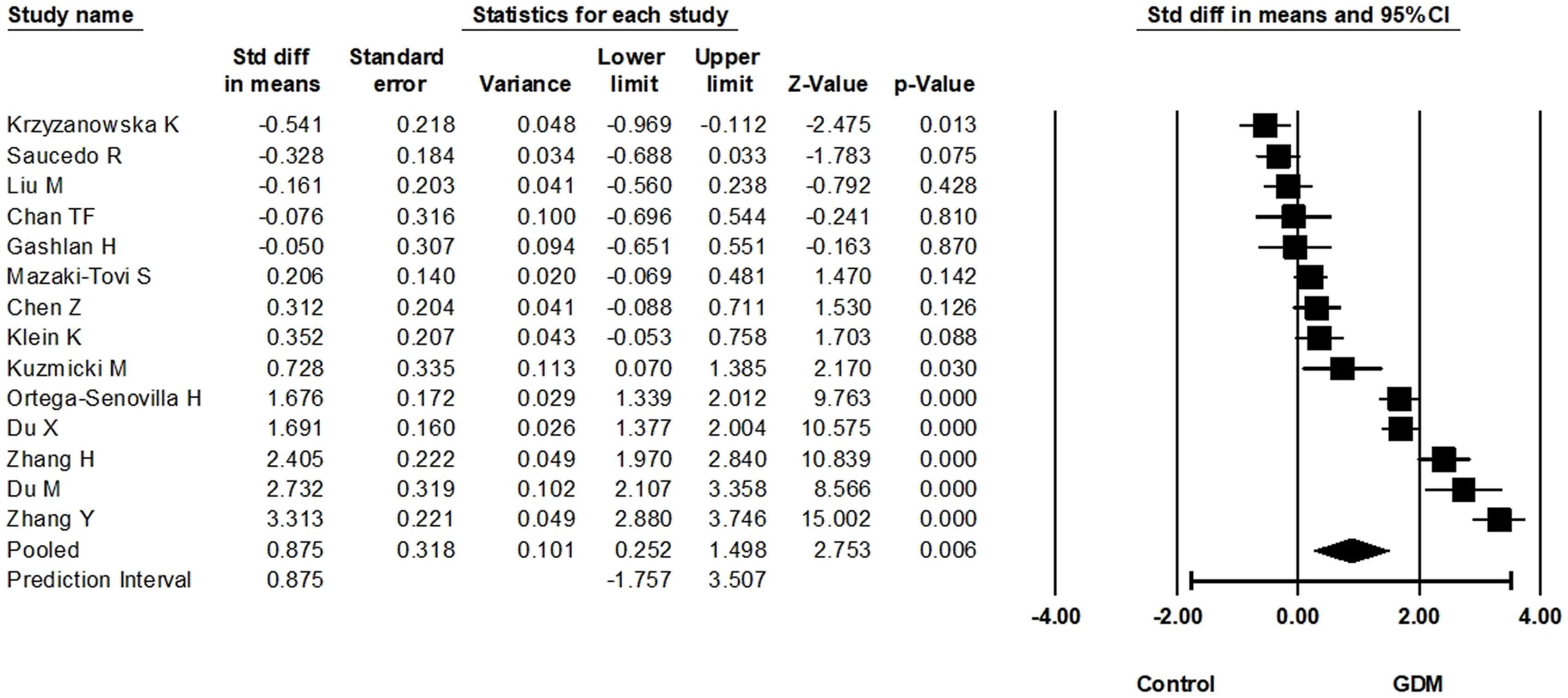
Forest plot of circulating RBP4 levels: gestational diabetes mellitus (GDM) compared to control at more than 28 weeks of gestation. Std diff in means: standardized mean difference; CI: Confidence intervals.

In total, 14 eligible studies compared circulating RBP4 at >28 weeks of gestation and reported the corresponding RBP4 levels (870 GDM cases vs. 901 non-GMD controls). Based on these, circulating RBP4 levels at >28 weeks of pregnancy were statistically higher in women with GDM compared to non-GDM controls (SMD: 0.875; 95% CI: 0.252–1.498; *p* = 0.006) (Figure 5). Considerable heterogeneity was noted among these studies (*I*^2^ = 97%), suggesting potential differences in the true effect sizes among the populations under investigation. Furthermore, removal of the study with skewed data (24) slightly increased the SMD to 0.984 (95% CI: 0.348–1.620). A one-study-removed analysis was also performed, as presented in Supplementary Figure S2.5.

### 3.7 Sub-group analysis

During the first trimester, sub-group analyses were completed for GDM diagnosis, RBP4 measurement method/assay, and country of study (Supplementary Table S2.1). Regarding the applied GDM diagnostic criteria, only one sub-group (IADPSG criteria) had more than one study in; in this group, the effect estimate was increased (SMD: 0.347, 95% CI: 0.073–0.621), but so too was the degree of heterogeneity (*I*^2^ = 85.4%). For RBP4 measurement, there was also only one subgroup with more than one study included. Three studies used an R&D Systems ELISA (SMD: 0.305, 95% CI 0.203–0.406) which reduced the *I*^2^ in that sub-group to 0%. Similarly for country in which included studies were conducted, it was only studies from China that constituted a group including multiple studies; the effect estimate was larger (SMD: 0.473, 95% CI: 0.237–0.708) in this sub-group, but the *I*^2^ was practically unchanged.

For 24–28 weeks of gestation, the sub-group analysis based upon the applied GDM diagnostic criteria identified four sub-groups that contained more than one study (Supplementary Table S2.1). As such, studies using the ADA, or Carpenter-Coustan, or 4th Workshop conference criteria were grouped together based on the applied GDM diagnostic cut-offs/criteria specified in the corresponding papers (7 studies, 1894 participants). For these, the statistical effect was lost, whilst the *I*^2^ was reduced to 63.5%. For the sub-group of studies using the IADPSG, or FIGO, or People’s Republic of China Health Industry Standards criteria (4 studies, 931 participants), the SMD increased to 1.402 (95% CI: 0.084 to 2.721) and so too did the *I*^2^ (98.5%). When the studies using the WHO criteria were grouped there was still considerable heterogeneity (93.0%), and the statistical effect was lost. Finally, for the two studies using the NDDG GDM criteria, the effect estimate retained statistical significance (SMD: 1.198, 95% CI: 0.696–1.699), whilst heterogeneity was reduced to an amount that may not be important (*I*^2^ = 32.1%). When RBP4 measurement method/assay was sub-grouped, three sub-groups were created (Supplementary Table S2.1). The sub-groups which used either an R&D Systems (five studies; SMD: 1.151, 95% CI: 0.042–2.260) or a Phoenix Pharmaceuticals (five studies; SMD: 0.699, 95% CI: 0.167–1.232) ELISA retained statistical effects, but with considerable heterogeneity (*I*^2^ = 98.1 and 88.3%, respectively). For the third sub-group which used an Immundiagnostik AG ELISA, the statistical effect estimate was lost, whilst there was still evidence of substantial heterogeneity (*I*^2^ = 78.8%). Finally, for sub-group analysis based upon country in which included studies were conducted, only two countries had more than one study, namely China (8 studies, SMD: 1.001; *I*^2^ = 97.6%) and Poland (2 studies, SMD: 0.922; *I*^2^ = 85.8%). A considerable degree of heterogeneity was apparent in both these sub-groups, while a statistical effect was retained for the studies from China only (Supplementary Table S2.1).

When sub-group analysis was completed based upon the applied GDM diagnostic criteria for the >28 weeks of gestation timepoint (Supplementary Table S2.1), a statistical effect was not retained for any of these sub-groups. The heterogeneity remained at a considerable level (*I*^2^ > 95%) for all but one of these sub-groups, namely the sub-group of studies that applied the WHO criteria for which the heterogeneity was reduced to a level that may not be important (3 studies; *I*^2^ = 34.7%). When RBP4 measurement methods/assays were sub-grouped, only two sub-groups were formed. For the four studies which used the R&D Systems ELISA (SMD: 2.337, 95% CI: 2.130–2.544) a statistical effect was retained, whereas for the two studies which used the Phoenix Pharmaceuticals ELISA the statistical effect was lost. Both these sub-groups demonstrated a considerable amount of heterogeneity (*I*^2^ ≥ 90%). Finally, based upon country in which included studies were conducted, sub-group analysis was possible only for China (six studies) and Austria (two studies). A statistical effect was retained for the studies from China (SMD: 1.708, 95% CI: 0.634–2.782), but not for the studies from Austria. Both these sub-groups had a considerable degree of heterogeneity (*I*^2^ > 88%).

### 3.8 Publication bias

Egger’s regression intercept test indicated that publication was not present at 24–28 weeks of gestation (*t* = 1.3, *p* = 0.2) (Supplementary Figure S2.6) nor at >28 weeks of gestation (*t* = 0.2, *p* = 0.8) (Supplementary Figure S2.7).

## 4 Discussion

The pathogenesis of GDM remains a subject of intense research interest due to the increasing GDM prevalence and the potential significant health implications for both mothers and offspring. In this context, recent research has further focused on novel factors (e.g., circulating adipokines such as RBP4) which appear implicated in the pathogenesis of GDM and may be utilized as GDM biomarkers (59). Therefore, this systematic review and meta-analysis aimed to offer up-to-date, comprehensive evidence on the relationship between circulating RBP4 levels and GDM at various timepoints across the pregnancy. The present meta-analyses included data from seven eligible studies examining circulating RBP4 levels in the first trimester, 19 studies at 24–28 weeks, and 13 studies at >28 weeks of pregnancy. Overall, the results showed statistically higher RBP4 levels in women with GDM compared to non-GDM controls at these different pregnancy timepoints.

Indeed, such a statistical difference in the circulating RBP4 levels was evident during the first trimester when women with and without GDM were compared. This finding suggests that circulating RBP4 levels in early pregnancy may be an early biomarker for GDM; although, the limited number of eligible existing studies for this early pregnancy timepoint warrants caution in interpreting this finding. Nevertheless, this is in accord to that from a previous meta-analysis from Wu et al. (13) on the association between RBP4 levels in early pregnancy and GDM risk. However, the paucity of relevant data for this pregnancy trimester/timepoint was also noted in this previous meta-analysis, together with potential ethnic-related differences; hence, further research is clearly required to determine if circulating RBP4 has potential as a GDM-related biomarker during the first trimester.

The present meta-analysis also revealed statistically higher circulating RBP4 levels in GDM cases compared to non-GDM controls at 24–28 weeks of gestation. The noted moderate effect size during this pregnancy period suggests that such elevated circulating RBP4 levels may be associated to GDM. This finding is in accord with previous research indicating the potential role of RBP4 in insulin resistance and glucose metabolism regulation after the first trimester of pregnancy (13–15). Thus, monitoring circulating RBP4 levels in pregnant women during the second trimester could be further explored as a potential GDM biomarker.

Finally, at >28 weeks of pregnancy, our meta-analysis also revealed higher circulating RBP4 levels in patients with GDM compared to non-GDM controls. The relatively large effect size noted for this gestation period indicates a potential relationship between these RBP4 levels in late pregnancy and GDM. Indeed, it is plausible that elevated circulating RBP4 levels at this stage may reflect an intensified insulin-resistant state, a hallmark of GDM, although further research is also required to establish this link.

Collectively, the findings of the present systematic review and meta-analysis offer updated evidence, which is also in line with Huang et al. (14) who conducted the first reported meta-analysis of observational studies aiming to investigate the relationship between circulating RBP4 levels and GDM. Indeed, their data included a total of 14 studies with 884 women with GDM and 1,251 normoglycemic pregnant women. Similar to the present meta-analysis, their overall results showed that circulating RBP4 levels were significantly higher in women with GDM compared to the studied controls. However, their stratified results indicated that this significant difference was observed only in the second/third trimester and was limited to Asian populations. This may be, at least in part, attributed to the lower number of eligible studies analyzed by Huang et al. (14), whilst potential ethnic differences in circulating RBP4 levels in pregnancy and GDM merits further targeted research. Another meta-analysis by Hu et al. (15) also included 14 case–control studies on serum RBP4 levels and GDM risk, involving a total of 647 GDM cases and 620 controls. This showed that high serum RBP4 levels represent a risk factor for GDM, with a pooled SMD of 0.758 (95% CI: 0.387–1.128). Their subgroup analyses based on gestational age at blood sampling and diagnostic criteria were consistent with the overall results, supporting the hypothesis that elevated RBP4 is a modest independent risk factor for GDM. However, in contrast with our present findings, no association was found by Hu et al. between circulating RBP4 levels and GDM in the first trimester. This may be partly due to changing insulin resistance levels during pregnancy; however, it should be noted that our meta-analysis included seven studies which assessed circulating RBP4 levels during the first trimester, while only one such study was included in the analyses by Hu et al. (15), potentially reducing the reliability of their stratified analysis on this point. Finally, another meta-analysis (60) that focused on the association of leptin and RBP4 with GDM risk included six studies with a total of 2,715 participants and 841 cases of GDM. In that meta-analysis, serum RBP4 levels also showed a significant positive association with the overall GDM risk, and pregnant women with the highest serum RBP4 levels were 2.04-fold more prone to GDM than those with the lowest levels. However, as with our findings, significant heterogeneity of the included studies was also noted (60). Overall, the exiting evidence supports the association of higher circulating RBP4 levels during pregnancy in patients with GDM, whilst this association appears to be more consistent in later pregnancy stages (second/third trimester), as was also documented in the aforementioned previous meta-analyses (14, 15). While this growing evidence is promising, further research is still required to advance our understanding, validate previous findings, and better explore the clinical implications of circulating RBP4 in the context of GDM.

The present meta-analysis has several strengths, including a comprehensive study selection process, thorough risk of bias assessment, and a relatively large sample size of 32 included studies with 3,595 GDM cases and 4,544 non-GDM controls, which is larger than previous meta-analyses on this topic. Indeed, by including detailed temporal analyses at different (early, mid, and late) pregnancy stages, the present work adds to the understanding of the potential association between circulating RBP4 levels and GDM. Moreover, the performed sensitivity analyses, addressing skewed data and the impact of specific studies, enhance the robustness of the present findings. Finally, our systematic review and meta-analysis addresses and evaluates potential publication bias, contributing to the overall reliability of the reported results.

However, certain limitations of the present work should also be acknowledged. Firstly, the total number of existing eligible studies included in some analyses was limited, which may have affected the robustness of the results. In addition, the study designs, participant characteristics, and laboratory methods for measuring RBP4 varied among the included studies, contributing to the observed high heterogeneity which may affect the reliability of the meta-analysis results, whilst inconsistencies in how relevant data are reported across the included studies might affect the accuracy of the present meta-analysis. A meta-regression would have been useful to help explain the high degree of heterogeneity in the analyses, but this was not performed due to inconsistencies with how continuous variables were reported across the identified studies (i.e., not all studies reported all variables). Moreover, variation in the methods used to measure circulating RBP4 levels across the identified studies could impact on the comparability of the results. Notably, most of the included studies were retrospective case–control studies, thus causality could not be established, whilst this may also introduce bias. The generalizability of the findings may be also limited by the small sample sizes of some of the included eligible studies. Furthermore, the identified statistically significant differences between GDM and non-GMD pregnancies cannot be necessarily considered as clinically significant, particularly given the proximity of the lower bound CI to zero. As is also common in systematic reviews, the possibility of publication bias, where studies with significant findings are more likely to be published, may have introduced a bias regarding the eligible studies which are published and are subsequently included in the searched databases. Finally, although multiple established biomedical databases were searched, the present systematic review identified only articles with English-language abstracts and main text written in either English or Chinese, which may have introduced a potential language bias.

## 5 Conclusion

The present systematic review and meta-analysis offers updated and comprehensive data which suggest that circulating RBP4 levels measured at different pregnancy timepoints/stages are higher in patients with GDM compared to non-GDM controls. Taken together with previous findings, this suggests that circulating RBP4 could be considered as a potential biomarker associated with GDM. Given that circulating levels of RBP4 are not routinely measured in the clinical practice, it is plausible that standardizing the method/assay for measuring circulating RBP4 in routine practice and adding this measurement as part of the GDM risk assessment visit/protocol in the context of antenatal care may be helpful to promptly identify those at high risk. However, the scarcity of relevant data particularly for early pregnancy and the noted high study heterogeneity, as well as factors relating to variability in RBP4 measurement methods and GDM diagnostic criteria/protocols, highlight the need for additional research in this field. Particularly, prospective (including the first trimester) and large-scale cohort studies across diverse populations and with standardized measurements of circulating RBP4 are needed to validate the present findings and confirm the generalizability of existing evidence. Future studies should also explore the potential underlying biological mechanisms which may link RBP4 to GDM, considering key pathophysiologic factors, such as insulin resistance and obesity-related inflammation.

## Data availability statement

The original contributions presented in the study are included in the article/Supplementary material, further inquiries can be directed to the corresponding author.

## Author contributions

BL: Conceptualization, Data curation, Formal analysis, Investigation, Methodology, Project administration, Resources, Software, Validation, Visualization, Writing – original draft, Writing – review & editing. CK: Conceptualization, Data curation, Formal analysis, Investigation, Methodology, Project administration, Resources, Software, Supervision, Validation, Visualization, Writing – original draft, Writing – review & editing. LL: Data curation, Formal analysis, Methodology, Project administration, Software, Validation, Visualization, Writing – original draft, Writing – review & editing. ADav: Data curation, Writing – original draft, Writing – review & editing. ADal: Data curation, Formal analysis, Software, Writing – original draft, Writing – review & editing. KC: Data curation, Writing – original draft, Writing – review & editing. HR: Conceptualization, Data curation, Formal analysis, Investigation, Methodology, Project administration, Resources, Software, Supervision, Validation, Visualization, Writing – original draft, Writing – review & editing. IK: Methodology, Project administration, Resources, Software, Supervision, Validation, Visualization, Writing – original draft, Writing – review & editing, Conceptualization, Data curation, Formal analysis, Investigation.

## Footnotes

^1^ https://apps.automeris.io/wpd/
