# Supplementary material for: Retinol-binding protein 4 (RBP4) circulating levels and gestational diabetes mellitus: a systematic review and meta-analysis
Source: Front Public Health. 2024 Mar 12;12:1348970. doi: 10.3389/fpubh.2024.1348970 (PMC10964926; doi:10.3389/fpubh.2024.1348970)

**Supplementary Material**

# Supplementary Data

## PRISMA checklist

| **Section and Topic** | **Item #** | **Checklist item** | **Page where item is reported** |
| --- | --- | --- | --- |
| **TITLE** | | |  |
| Title | 1 | Identify the report as a systematic review. | 1 |
| **ABSTRACT** | | |  |
| Abstract | 2 | See the PRISMA 2020 for Abstracts checklist. | 1 |
| **INTRODUCTION** | | |  |
| Rationale | 3 | Describe the rationale for the review in the context of existing knowledge. | 2 |
| Objectives | 4 | Provide an explicit statement of the objective(s) or question(s) the review addresses. | 2 |
| **METHODS** | | |  |
| Eligibility criteria | 5 | Specify the inclusion and exclusion criteria for the review and how studies were grouped for the syntheses. | 3 |
| Information sources | 6 | Specify all databases, registers, websites, organisations, reference lists and other sources searched or consulted to identify studies. Specify the date when each source was last searched or consulted. | 2 |
| Search strategy | 7 | Present the full search strategies for all databases, registers and websites, including any filters and limits used. | 3 |
| Selection process | 8 | Specify the methods used to decide whether a study met the inclusion criteria of the review, including how many reviewers screened each record and each report retrieved, whether they worked independently, and if applicable, details of automation tools used in the process. | 3 |
| Data collection process | 9 | Specify the methods used to collect data from reports, including how many reviewers collected data from each report, whether they worked independently, any processes for obtaining or confirming data from study investigators, and if applicable, details of automation tools used in the process. | 3 |
| Data items | 10a | List and define all outcomes for which data were sought. Specify whether all results that were compatible with each outcome domain in each study were sought (e.g. for all measures, time points, analyses), and if not, the methods used to decide which results to collect. | 3 |
|  | 10b | List and define all other variables for which data were sought (e.g. participant and intervention characteristics, funding sources). Describe any assumptions made about any missing or unclear information. | 3 |
| Study risk of bias assessment | 11 | Specify the methods used to assess risk of bias in the included studies, including details of the tool(s) used, how many reviewers assessed each study and whether they worked independently, and if applicable, details of automation tools used in the process. | 3 |
| Effect measures | 12 | Specify for each outcome the effect measure(s) (e.g. risk ratio, mean difference) used in the synthesis or presentation of results. | 3 |
| Synthesis methods | 13a | Describe the processes used to decide which studies were eligible for each synthesis (e.g. tabulating the study intervention characteristics and comparing against the planned groups for each synthesis (item #5)). | 3 |
|  | 13b | Describe any methods required to prepare the data for presentation or synthesis, such as handling of missing summary statistics, or data conversions. | 3 |
|  | 13c | Describe any methods used to tabulate or visually display results of individual studies and syntheses. | 3 |
|  | 13d | Describe any methods used to synthesize results and provide a rationale for the choice(s). If meta-analysis was performed, describe the model(s), method(s) to identify the presence and extent of statistical heterogeneity, and software package(s) used. | 3 |
|  | 13e | Describe any methods used to explore possible causes of heterogeneity among study results (e.g. subgroup analysis, meta-regression). | 3 |
|  | 13f | Describe any sensitivity analyses conducted to assess robustness of the synthesized results. | 3 |
| Reporting bias assessment | 14 | Describe any methods used to assess risk of bias due to missing results in a synthesis (arising from reporting biases). | 3 |
| Certainty assessment | 15 | Describe any methods used to assess certainty (or confidence) in the body of evidence for an outcome. | 3 |
| **RESULTS** | | |  |
| Study selection | 16a | Describe the results of the search and selection process, from the number of records identified in the search to the number of studies included in the review, ideally using a flow diagram. | 3-4 |
|  | 16b | Cite studies that might appear to meet the inclusion criteria, but which were excluded, and explain why they were excluded. | 4 |
| Study characteristics | 17 | Cite each included study and present its characteristics. | 6-10 |
| Risk of bias in studies | 18 | Present assessments of risk of bias for each included study. | 4-5 |
| Results of individual studies | 19 | For all outcomes, present, for each study: (a) summary statistics for each group (where appropriate) and (b) an effect estimate and its precision (e.g. confidence/credible interval), ideally using structured tables or plots. | 6-8 |
| Results of syntheses | 20a | For each synthesis, briefly summarise the characteristics and risk of bias among contributing studies. | 5 |
|  | 20b | Present results of all statistical syntheses conducted. If meta-analysis was done, present for each the summary estimate and its precision (e.g. confidence/credible interval) and measures of statistical heterogeneity. If comparing groups, describe the direction of the effect. | 5, 11-12 |
|  | 20c | Present results of all investigations of possible causes of heterogeneity among study results. | 12 |
|  | 20d | Present results of all sensitivity analyses conducted to assess the robustness of the synthesized results. | 5, 11-12 |
| Reporting biases | 21 | Present assessments of risk of bias due to missing results (arising from reporting biases) for each synthesis assessed. | 4-5 |
| Certainty of evidence | 22 | Present assessments of certainty (or confidence) in the body of evidence for each outcome assessed. | 12 |
| **DISCUSSION** | | |  |
| Discussion | 23a | Provide a general interpretation of the results in the context of other evidence. | 12-13 |
|  | 23b | Discuss any limitations of the evidence included in the review. | 13 |
|  | 23c | Discuss any limitations of the review processes used. | 13 |
|  | 23d | Discuss implications of the results for practice, policy, and future research. | 13-14 |
| **OTHER INFORMATION** | | |  |
| Registration and protocol | 24a | Provide registration information for the review, including register name and registration number, or state that the review was not registered. | 2 |
|  | 24b | Indicate where the review protocol can be accessed, or state that a protocol was not prepared. | 2 |
|  | 24c | Describe and explain any amendments to information provided at registration or in the protocol. | N/A |
| Support | 25 | Describe sources of financial or non-financial support for the review, and the role of the funders or sponsors in the review. | 14 |
| Competing interests | 26 | Declare any competing interests of review authors. | 14 |
| Availability of data, code and other materials | 27 | Report which of the following are publicly available and where they can be found: template data collection forms; data extracted from included studies; data used for all analyses; analytic code; any other materials used in the review. | N/A |

## Search strategy as applied for each additional searched database

### Cinahl

| # | Query |
| --- | --- |
| 1 | (retinol binding protein 4) OR (retinol-binding protein-4) OR (retinol binding protein-4) OR RBP4 OR (RBP 4) OR (RBP-4) |
| 2 | (MH "Pregnancy") OR pregnan* |
| 3 | 1 AND 2 |

### Emcare and Embase (via Ovid)

| # | Query |
| --- | --- |
| 1 | exp retinol binding protein/ or retinol binding protein 4.mp. or retinol-binding protein-4.mp. or retinol binding protein-4.mp. or RBP4.mp. or RBP 4.mp. or RBP-4.mp. |
| 2 | exp pregnancy/ or pregnan*.af. |
| 3 | 1 AND 2 |

### Scopus

| (TITLE-ABS-KEY ("retinol binding protein 4" OR "retinol-binding protein-4" OR "retinol binding protein-4" OR "RBP4" OR "RBP 4" OR "RBP-4"))  AND  (TITLE-ABS-KEY ("pregnan*")) |
| --- |

### Web of Science

| # | Query |
| --- | --- |
| 1 | retinol binding protein (Topic) or "retinol-binding protein-4" OR "retinol binding protein-4" OR "RBP4" OR "RBP 4" OR "RBP-4" (All Fields) |
| 2 | pregnancy (Topic) or "pregnan*” (All Fields) |
| 3 | 1 AND 2 |

##

## Circulated Retinol-binding protein 4 (RBP4) levels per timepoint of gestation reported by the included eligible studies.

| **RBP4 data during 1st trimester** | | | **RBP4 data at 24-28 weeks of gestation** | | | **RBP4 data >28 weeks of gestation** | | |
| --- | --- | --- | --- | --- | --- | --- | --- | --- |
| **Study** | **GDM** | **Controls** | **Study** | **GDM** | **Controls** | **Study** | **GDM** | **Controls** |
| Abetew DF *et al.* [41] | 47.13 ± 30.02 μg/mL | 41.14 ± 21.29 μg/mL | Chan TF *et al.* [42] | 42.4 ± 13.8 ng/mL | 32 ± 8.7 ng/mL | Chan TF *et al.* [42] | 30.1 ± 11 ng/mL | 30.9 ± 10 ng/mL |
| Fruscalzo A *et al.* [32] | 24.78 ± 6.93 μmol/L | 27.72 ± 8.4 μmol/L | Du X *et al.* [44] | 41.1 ± 7.26 μg/mL | 30.55 ± 5.54 μg/mL | Chen Z *et al.* [31] | 29.44 ± 7.23 μg/L | 26.92 ± 8.96 μg/L |
| Wu P *et al.* [13] | 25.57 ± 5.98 μg/mL | 23.76 ± 5.66 μg/mL | Ping F *et al.* [45] | 21.53 ± 5.69 μg/mL | 20.84 ± 4.31 μg/mL | Du M *et al.* [43] | 39.08 ± 8.29 μg/mL | 21.42 ± 3.85 μg/mL |
| Yuan X *et al.* [54] | 32.21 ± 5.27 μg/mL | 28.45 ± 3.99 μg/mL | Gashlan H *et al.* [46] | 2.22 ± 1.45 ng/mL | 3.86 ± 1.62 ng/mL | Du X *et al.* [44] | 43.5 ± 7.13 μg/mL | 31.18 ± 7.73 μg/mL |
| Hou W *et al.* [18] | 37 (30-45) mg/L | 34 (28-39) mg/L | Jia X *et al.* [47] | 33.08 ± 20.28 μg/mL | 30.79 ± 21.51 μg/mL | Gashlan H *et al.* [46] | 3.16 ± 0.66 ng/mL | 3.2 ± 0.97 ng/mL |
| Jin C *et al.* [19] | 18 (10.3-31.6) μg/mL | 14.1 (9.6-21.8) μg/mL | Khovidhunkit W *et al.* [33] | 36 ± 10.4 μg/mL | 34.6 ± 6.7 μg/mL | Klein K *et al.* [49] | 20.3 ± 7.2 mg/L | 17.9 ± 6.1 mg/L |
| Nanda S *et al.* [20] | 13,809 (11,410- 15821) ng/mL | 12,605 (10,492-15.298) ng/mL | Kim SH *et al.* [48] | 39.1 ± 6.3 μg/mL | 30 ± 10 μg/mL | Liu M *et al.* [51] | 49119.23 ± 13782.69 μg/mL | 51463.17 ± 15316.72 μg/mL |
| Francis E *et al.* [39] | Not reported | Not reported | Klein K *et al.* [49] | 18 ± 3.7 mg/L | 16.9 ± 5.1 mg/L | Ortega-Senovilla H *et al.* [28] | 124.58 ± 40.09 μg/mL | 59.76 ± 37 μg/mL |
|  |  |  | Lewandowski K C *et al.* [50] | 53.9 ± 17.9 μg/mL | 29.7 ± 13.9 μg/mL | Saucedo R *et al.* [40] | 4.7 ± 1.9 μg/mL | 5.3 ± 1.76 μg/mL |
|  |  |  | Maghbooli Z *et al.* [52] | 0.49 ± 0.13 μg/mL | 0.37 ± 0.14 μg/mL | Zhang H *et al.* [55] | 62.45 ± 7.86 mg/L | 45.48 ± 6.15 mg/L |
|  |  |  | Su YX *et al.* [53] | 41.6 ± 12.2 mg/L | 34.5 ± 9.8 mg/L | Zhang Y *et al.* [56] | 51.79 ± 4.64 mg/L | 39.35 ± 3.59 mg/L |
|  |  |  | Tepper BJ *et al.* [35] | 1.2 ± 0.34 μmol/L | 1.2 ± 0.32 μmol/L | Krzyzanowska K *et al.* [24] | 6.8 (3.3-14.4) μg/mL | 11.3 (7.8-19.9) μg/mL |
|  |  |  | Zhang H *et al.* [55] | 49.28 ± 4.83 mg/L | 36.82 ± 3.12 mg/L | Kuzmicki M *et al.* [22] | 62.4 45.6-87.4 mg/L | 42.9 (32.9-58.5) mg/L |
|  |  |  | Zhaoxia L *et al.* [57] | 22.9 ± 3.09 μg/mL | 17.9 ± 3.91 μg/mL | Mazaki-Tovi S *et al.* [25] | 10317.46 ± 3264.46 ng/mL | 9726.86 ± 2450.45 ng/mL |
|  |  |  | Zhu JP *et al.* [58] | 42.92 ± 7.51 mg/L | 40.52 ± 6.85 mg/L | Francis E *et al.* [39] | Not reported | Not reported |
|  |  |  | Gorkem U *et al.* [21] | 15.29 (10.08-19.63) mg/mL | 14.91 (10.64-31.16) mg/mL |  |  |  |
|  |  |  | Jin C *et al.* [19] | 18 (11.3-29.1) μg/L | 15.4 (9.6-21.8) μg/L |  |  |  |
|  |  |  | Kuzmicki M *et al.* [22] | 58.1 (48.5-82.7) mg/L | 51 (43.2-67.7) mg/L |  |  |  |
|  |  |  | Skvarca A *et al.* [23] | 15 (10.5-18.75) mg/L | 16 (12-24) mg/L |  |  |  |
|  |  |  | Francis E *et al.* [39] | Not reported | Not reported |  |  |  |

# Supplementary Figures and Tables

## Summary of effect estimates and heterogeneity from sub-group analyses by pregnancy stage for GDM diagnosis method, RBP4 measurement method, and country where study was completed.

| **Trimester** | **Sub-analysis** | **Sub-group** | **Trials (N)** | **Effect estimate SMD (95% CI)** | **I^2^** |
| --- | --- | --- | --- | --- | --- |
| **1^st^ trimester** | GDM Diagnostic Criteria^§^ | IADPSG | 5 (1990) | 0.347 (0.073 to 0.621)* | 85.4% |
|  |  | ADA | 1 (360) | 0.232 (0.024 to 0.439)* | NA |
|  |  | WHO | 1 (300) | 0.244 (-0.040 to 0.528) | NA |
|  | RBP4 Measurement | R&D Systems ELISA | 3 (1626) | 0.305 (0.203 to 0.406)*** | 0% |
|  |  | Automatic analyser using commercial kit (Wako Pure Chemical Industries, Osaka, Japan) | 1 (359) | 0.869 (0.618 to 1.119)* | NA |
|  |  | Not specified kit/assay | 1 (269) | 0.377 (0.136 to 0.618)** | NA |
|  |  | Immundiagnostik AG ELISA | 1 (300) | 0.244 (-0.040 to 0.528) | NA |
|  |  | In-house ELISA | 1 (96) | -0.370 (-0.798 to 0.058) | NA |
|  | Country | China | 4 (1894) | 0.473 (0.237 to 0.708)*** | 80.1% |
|  |  | Italy | 1 (96) | -0.370 (-0.798 to 0.058) | NA |
|  |  | UK | 1 (300) | 0.244 (-0.040 to 0.528) | NA |
|  |  | USA | 1 (360) | 0.232 (0.024 to 0.439)* | NA |
|  |  | **Overall effect estimate** | **7 (2647)** | **0.322 (0.126 to 0.517)***** | **80.0%** |
| **24-28 weeks** | GDM Diagnostic Criteria^§^ | ADA or Carpenter-Coustan or 4th Workshop Conference on GDM criteria | 7 (1638) | 0.174 (-0.072 to 0.420) | 63.5% |
|  |  | IADPSG or FIGO or People’s Republic of China Health Industry Standards | 4 (931) | 1.402 (0.084 to 2.721)* | 98.5% |
|  |  | WHO | 3 (215) | 0.279 (-0.952 to 1.511) | 93.0% |
|  |  | NDDG | 2 (110) | 1.198 (0.696 to 1.699)*** | 32.1% |
|  |  | German & Austrian Society for Diabetes (modified Carpenter Coustan criteria) | 1 (101) | 0.257 (-0.147 to 0.661) | NA |
|  |  | O’Sullivan & Mahan | 1 (192) | 0.887 (0.590 to 1.184)*** | NA |
|  |  | Not specified | 1 (531) | 0.339 (0.158 to 0.521)*** | NA |
|  | RBP4 Measurement | R&D Systems ELISA | 5 (1124) | 1.151 (0.042 to 2.260)* | 98.1% |
|  |  | Phoenix Pharmaceuticals ELISA | 5 (1333) | 0.699 (0.167 to 1.232)** | 88.3% |
|  |  | Immundiagnostik AG ELISA | 4 (272) | 0.297 (-0.315 to 0.909) | 78.8% |
|  |  | In-house ELISA | 1 (121) | 0.639 (0.273 to 1.004)*** | NA |
|  |  | AdipoGen ELISA | 1 (192) | 0.887 (0.590 to 1.184)*** | NA |
|  |  | DRG Instruments ELISA | 1 (101) | 0.257 (-0.147 to 0.661) | NA |
|  |  | Elabscience ELISA | 1 (44) | -1.075 (-1.713 to -0.438) | NA |
|  |  | Not Specified | 1 (531) | 0.339 (0.158 to 0.521)*** | NA |
|  | Country | China | 8 (2723) | 1.001 (0.424 to 1.579)*** | 97.6% |
|  |  | Poland | 2 (171) | 0.922 (-0.180 to 2.023) | 85.8% |
|  |  | Austria | 1 (101) | 0.257 (-0.147 to 0.661) | NA |
|  |  | Saudi Arabia | 1 (44) | -1.075 (-1.713 to -0.438) | NA |
|  |  | Slovenia | 1 (55) | -0.341 (-0.875 to 0.194) | NA |
|  |  | South Korea | 1 (19) | 1.103 (0.137 to 2.069)* | NA |
|  |  | Taiwan | 1 (40) | 0.902 (0.251 to 1.552)** | NA |
|  |  | Iran | 1 (192) | 0.887 (0.590 to 1.184)*** | NA |
|  |  | Thailand | 1 (193) | 0.139 (-0.305 to 0.583) | NA |
|  |  | Turkey | 1 (158) | -0.105 (-0.417 to 0.207) | NA |
|  |  | USA | 1 (22) | 0.183 (-0.658 to 1.024) | NA |
|  |  | **Overall effect estimate** | **19 (3718)** | **0.628 (0.290 to 0.966)***** | **95.0%** |
| **>28 weeks** | GDM Diagnostic Criteria^§^ | WHO | 3 (287) | 0.253 (-0.088 to 0.594) | 34.7% |
|  |  | ADA or Carpenter-Coustan or 4th Workshop Conference on GDM criteria | 3 (391) | 0.273 (-1.166 to 1.712) | 97.8% |
|  |  | IADPSG or FIGO | 3 (638) | 1.613 (-0.186 to 3.411) | 98.5% |
|  |  | NDDG | 2 (116) | 1.328 (-1.424 to 4.080) | 97.4% |
|  |  | Obstetrics and Gynecology Chinese Medical Association | 1 (140) | 2.405 (1.970 to 2.840)*** | NA |
|  |  | German & Austrian Society for Diabetes (modified Carpenter Coustan criteria) | 1 (101) | 0.352 (-0.053 to 0.758) | NA |
|  |  | Not specified | 1 (98) | 0.312 (-0.088 to 0.711) | NA |
|  | RBP4 Measurement | R&D Systems ELISA | 4 (757) | 2.337 (2.130 to 2.544)*** | 92.0% |
|  |  | Phoenix Pharmaceuticals ELISA | 2 (125) | 0.068 (-1.174 to 1.310) | 90.0% |
|  |  | AdipoGen ELISA | 1 (184) | 1.676 (1.339 to 2.012)*** | NA |
|  |  | Cusabio Biotech ELISA | 1 (97) | -0.161 (-0.560 to 0.238) | NA |
|  |  | DRG Instruments ELISA | 1 (101) | 0.352 (-0.053 to 0.758) | NA |
|  |  | Elabscience ELISA | 1 (44) | -0.050 (-0.651 to 0.551) | NA |
|  |  | Immundiagnostik AG ELISA | 1 (40) | -0.076 (-0.696 to 0.544) | NA |
|  |  | Millipore Corporation ELISA | 1 (205) | 0.206 (-0.069 to 0.481) | NA |
|  |  | Not specified ELISA | 1 (98) | 0.312 (-0.088 to 0.711) | NA |
|  |  | RIA using Phoenix Pharmaceuticals reagents | 1(120) | -0.328 (-0.688 to 0.033) | NA |
|  | Country | China | 6 (952) | 1.708 (0.634 to 2.782)*** | 97.5% |
|  |  | Austria | 2 (188) | -0.091 (-0.966 to 0.784) | 88.7% |
|  |  | Mexico | 1 (120) | -0.328 (-0.688 to 0.033) | NA |
|  |  | Poland | 1 (38) | 0.728 (0.070 to 1.385)* | NA |
|  |  | Saudi Arabia | 1 (44) | -0.050 (-0.651 to 0.551) | NA |
|  |  | Spain | 1 (184) | 1.676 (1.339 to 2.012)*** | NA |
|  |  | Taiwan | 1 (40) | -0.076 (-0.696 to 0.544) | NA |
|  |  | USA | 1 (205) | 0.206 (-0.069 to 0.481) | NA |
|  |  | **Overall effect estimate** | **14 (1771)** | **0.875 (0.252 to 1.498)**** | **97.0%** |
| **Abbreviations**: ADA: American Diabetes Association; ELISA: enzyme-linked immunosorbent assay; FIGO: International Federation of Gynecology and Obstetrics; GDM: gestational diabetes mellitus; I^2^: statistical measure of study heterogeneity; IADPSG: International Association of Diabetes and Pregnancy Study Groups; N: number of participants in analysis; NA: Not Applicable; NDDG: National Diabetes Data Group; RBP4: retinol-binding protein 4; RIA: radioimmunoassay; SMD: standardised mean difference; Trials: number of studies in analysis; WHO: World Health Organization. ^§^: Studies which applied the same criteria/cut-offs for the diagnosis of GDM, as specified in the corresponding paper, were grouped together for this sub-group analysis; *: P ≤ 0.05; **: P ≤ 0.01; ***: P ≤ 0.001 | | | | | |

## Risk of bias assessment according to the Risk of Bias Assessment Tool for Nonrandomized Studies (RoBANS) of all eligible studies included in the present systematic review.


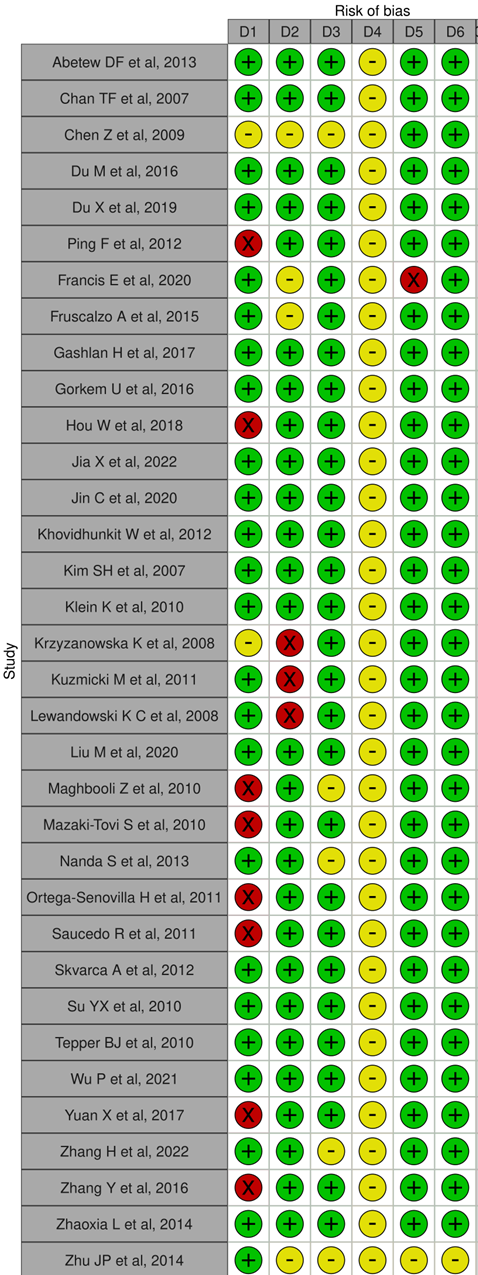


**Legend:** D1: selection of participants; D2: confounding variables; D3: exposure measurement; D4: blinding of outcome assessment; D5: incomplete outcome data; D6: selective outcome reporting; green: low risk of bias; yellow: unclear risk of bias; red: high risk of bias.

## One study removed analysis in the first pregnancy trimester.


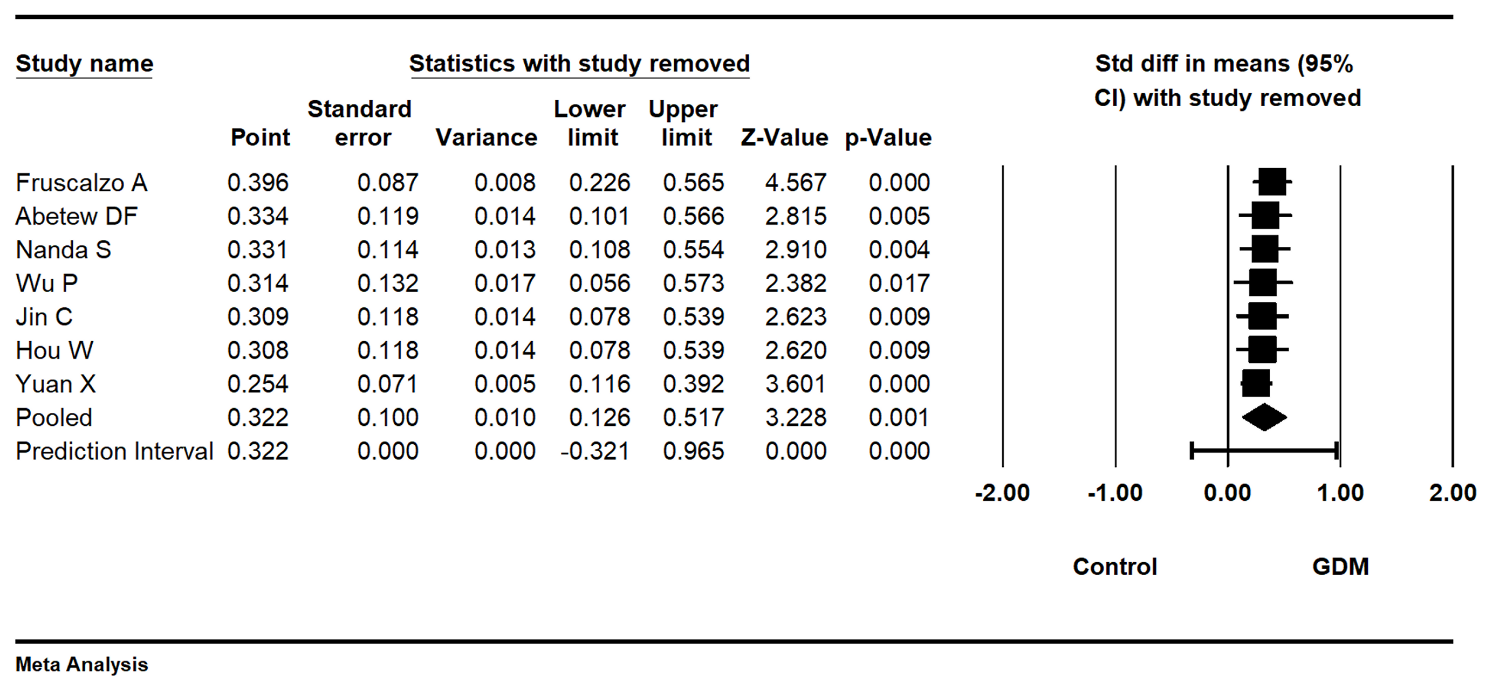


## One study removed analysis at 24-28 weeks of gestation.


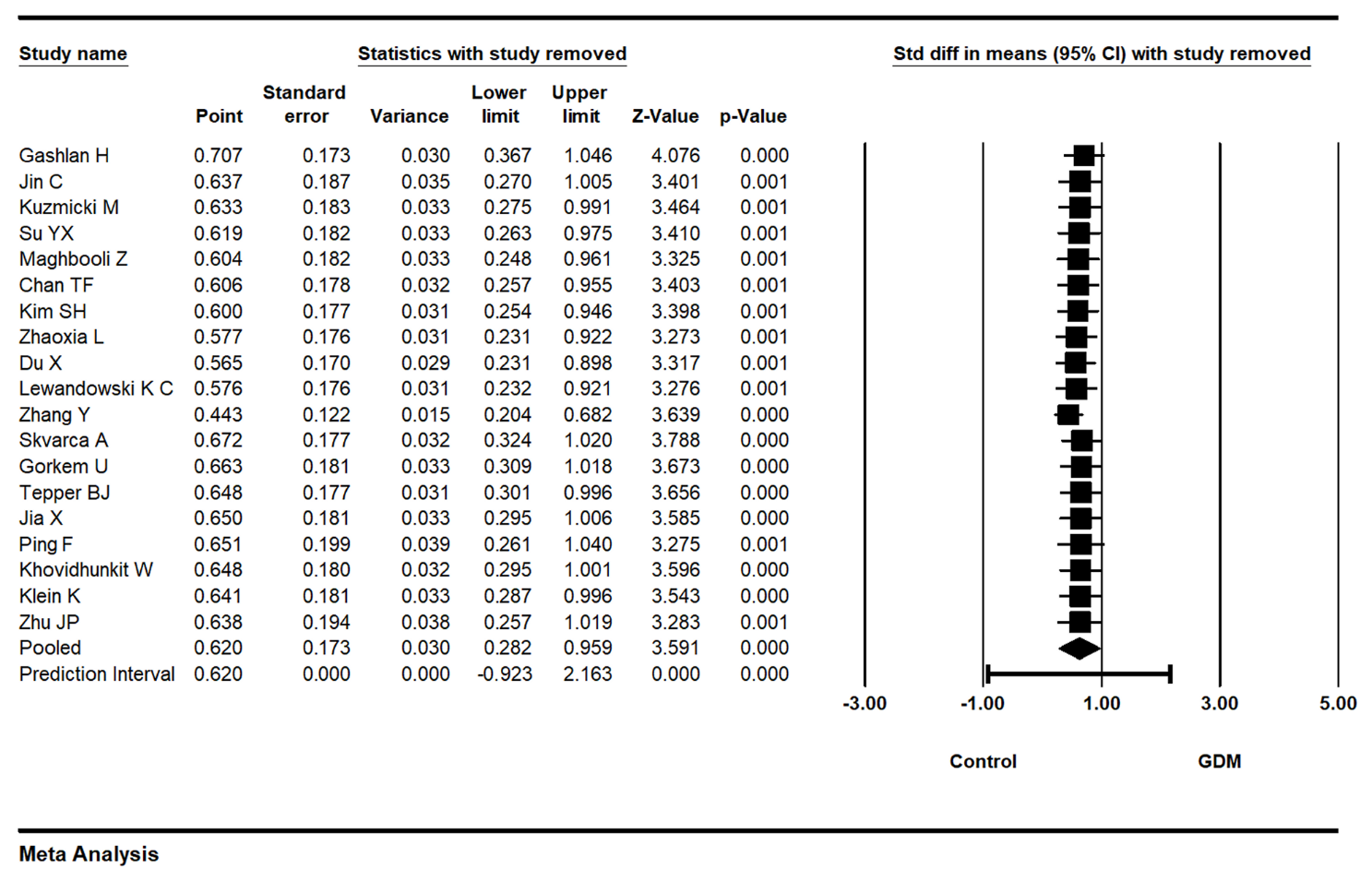


## One study removed analysis at more than 28 weeks of gestation.


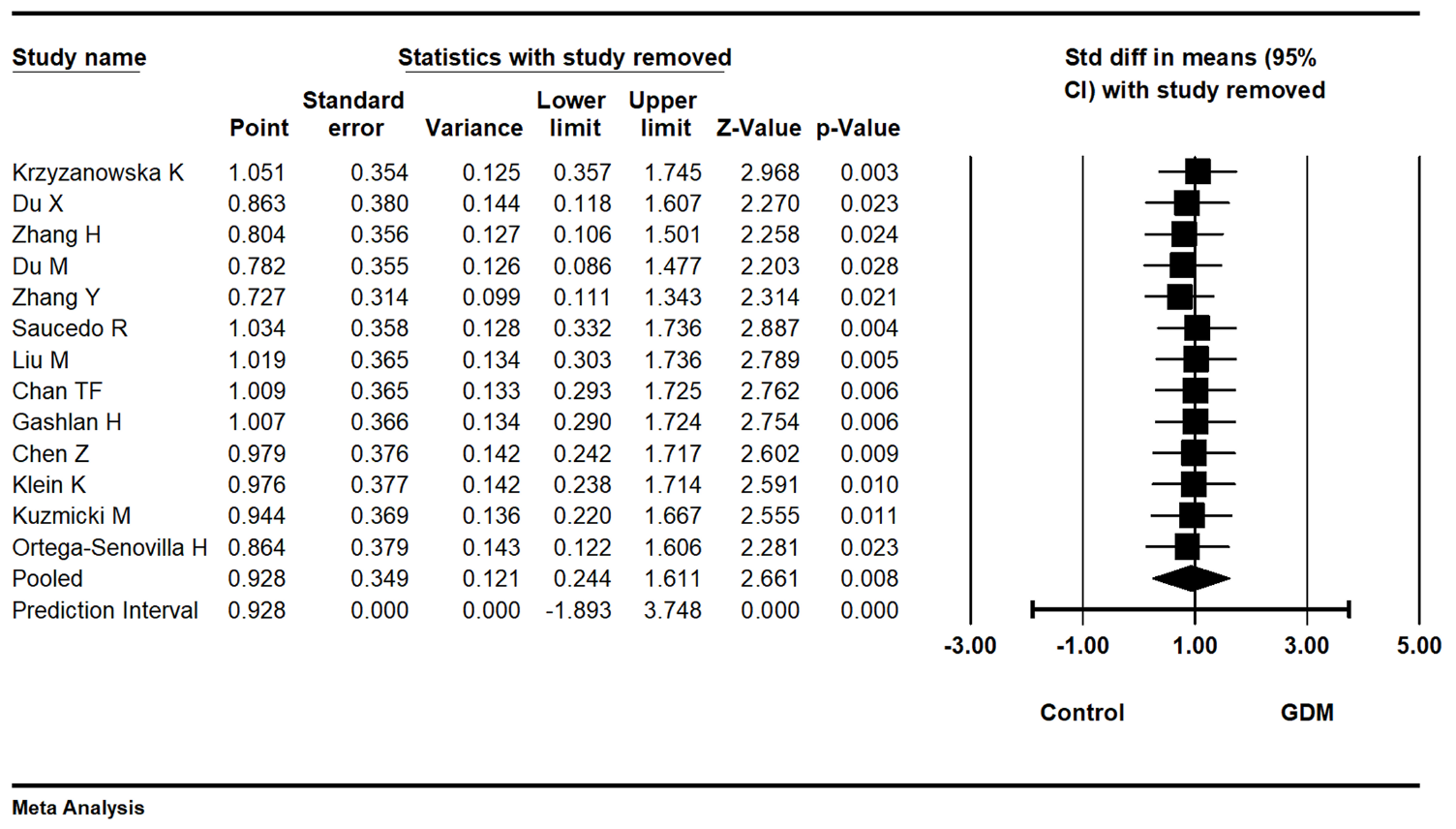


## Publication bias funnel plot at 24-28 weeks of gestation.


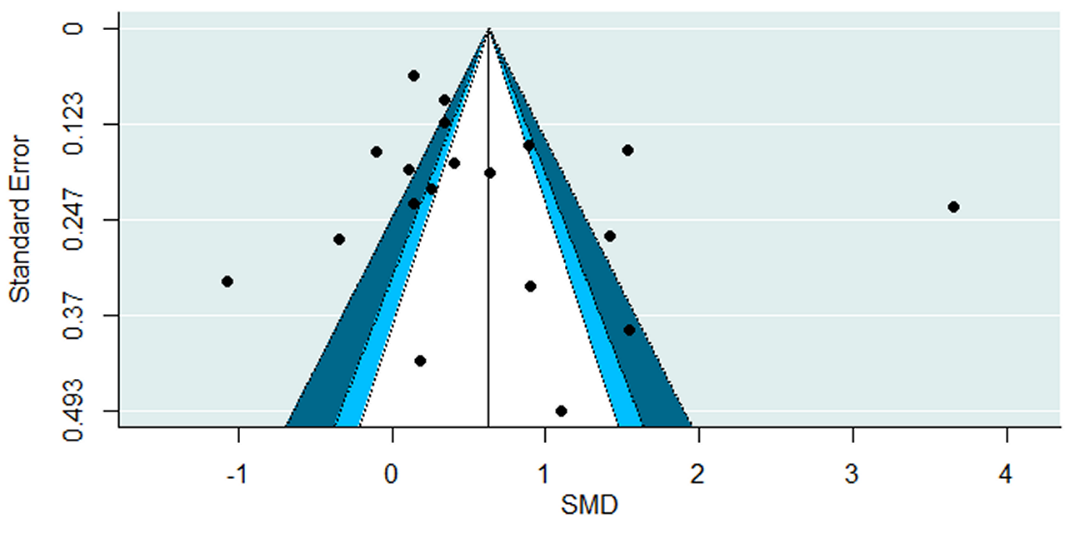


## Publication bias funnel plot at >28 weeks of gestation.


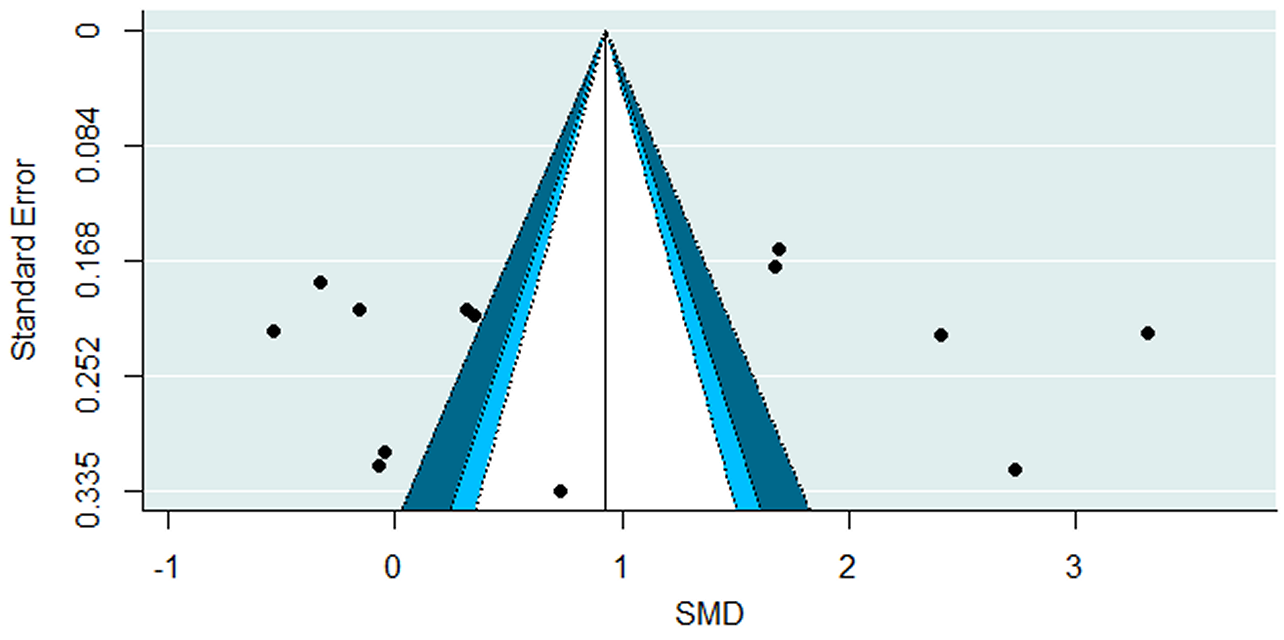

Supplement: Supplementary file 1 [file Data_Sheet_1.docx]
